# Supplementary material for: Dengue transmission dynamics prediction by combining metapopulation networks and Kalman filter algorithm
Source: PLoS Negl Trop Dis. 2023 Jun 7;17(6):e0011418. doi: 10.1371/journal.pntd.0011418 (PMC10281582; doi:10.1371/journal.pntd.0011418)
Supplement: S1 Text Method — (DOC) [file pntd.0011418.s001.doc]

**Supporting Information**

**Dengue transmission dynamics prediction by combining metapopulation networks and Kalman filter algorithm**

Qinghui Zeng ^1^, Xiaolin Yu ^1^, Haobo Ni ^1^, Lina Xiao ^1^, Ting Xu ^1^, Haisheng Wu ^1^, Yuliang Chen ^2^, Hui Deng ^3^, Yingtao Zhang ^4^, Sen Pei ^5, *^, Jianpeng Xiao ^6, *^, Pi Guo ^1, 7, *^

^1^ Department of Preventive Medicine, Shantou University Medical College, No. 22 Xinling Road, Shantou 515041, China

^2^ Department of Medical Quality Management, Nanfang Hospital, Guangzhou, 510515, China

^3^ Institute of Vector Control, Guangdong Provincial Center for Disease Control and Prevention, Guangzhou 511430, Guangdong, China

^4^ Institute of Infectious Disease Control and Prevention, Guangdong Provincial Center for Disease Control and Prevention, Guangzhou 511430, Guangdong, China

^5^ Department of Environmental Health Sciences, Mailman School of Public Health, Columbia University, New York, NY 10032, USA

^6^ Guangdong Provincial Institute of Public Health, Guangdong Provincial Center for Disease Control and Prevention, Guangzhou 511430, China

^7^ Guangdong Provincial Key Laboratory of Infectious Diseases and Molecular Immunopathology, Shantou 515041, China

^*^ **Corresponding author:**

[sp3449@cumc.columbia.edu](mailto:sp3449@cumc.columbia.edu) (SP), [xiaojp@gdiph.org.cn](mailto:xiaojp@gdiph.org.cn) (JX), [pguo@stu.edu.cn](mailto:pguo@stu.edu.cn) (PG)

**Text S1. Method.**

1. **The association of climate, mosquito vectors, and population movements with the occurrence of dengue fever**

Time-series: weekly observations of dengue fever, temperature, Mosquito Oviposition Index (MOI), and population movements for each prefecture-level city in Guangdong Province from 2018 to 2020 were presented separately in S6A Fig. To identify the mechanisms driving the dengue epidemic, we used empirical dynamics modeling (EDM) [1, 2], a mechanistic, equation-free, data-driven approach that accounts for the environmental dependence of ecological drivers. EDM is based on reconstructing the system dynamics in a time series without assuming fixed relationships. If the relationship between variables depends on the environment, then the relationship between variables can change over time. EDM constructs attractors to empirically derive the dynamic relationship from time-series observations. Using the above method, we present the cross mapping skill given by EDM in the figure. Except for individual cities, the cross mapping skill of most cities is positive, reflecting that climate, mosquito vector, and population movement all show a positive correlation with the occurrence of dengue fever. Using the above method, we presented the cross mapping skill given by EDM in S6B Fig. Except for individual cities, the cross mapping skill of most cities was positive, reflecting that the time series of climate, mosquito vector, and population movement showed a positive correlation with the occurrence of dengue fever.

1. **Analysis of the intercity population mobility patterns**

In the population mobility data between cities in Guangdong province, China, most population movements occured between neighboring cities. We divided pairs of cities into adjacent and non-adjacent categories and then ploted the distribution of the number of travelers in each category (S4 Fig). A high degree of heterogeneity existed in cross-city migration, spaning several orders of magnitude. Population mobility is generally higher between adjacent cities than between non-adjacent cities. Therefore, the main effect of the population mobility model on the spread of the epidemic may be concentrated in the neighboring cities.

1. **Temperature and mosquito vector-driven SIR model for dengue fever**

As a mosquito-borne disease, Dengue fever must be transmitted with the medium of mosquitoes. In addition, the mosquito-borne disease is bidirectional and can be transmitted from infected mosquitoes to susceptible individuals or from infected individuals to susceptible mosquitoes. Therefore, we used a temperature and mosquito-vector-driven SIR model of dengue fever to simulate dengue transmission for the isolated model. This model was validated in a previous study [3]. During the course of disease transmission, each individual may progress across the compartments, with the rate illustrated by these ordinary differential equations:

$$\frac{dS_{M}}{dt}=-\frac{\tau(t)\beta_{0}S_{M}I_{H}}{N_{H}}-\alpha S_{M}+\mu_{b}(t)(S_{M}+(1-U)I_{M})-\mu_{d}S_{M}$$

$$\frac{dI_{M}}{dt}=\frac{\tau(t)\beta_{0}S_{M}I_{H}}{N_{H}}+\alpha S_{M}+\mu_{b}(t)UI_{M}-\mu_{d}I_{M}$$

$$\frac{dS_{H}}{dt}=\frac{\tau(t)\beta_{0}S_{H}I_{M}}{N_{H}}$$

$$\frac{dI_{H}}{dt}=\frac{\tau(t)\beta_{0}S_{H}I_{M}}{N_{H}}-\frac{I_{H}}{D}$$

$$\beta(t)=\tau(t)\beta_{0}$$

$$N_{H}(t)=S_{H}(t)+I_{H}(t)+R_{H}(t)$$

where $S_{M}$ denotes the number of susceptible mosquitoes, $I_{M}$ represents the number of infected mosquitoes. $S_{H}$ is the number of susceptible humans, $I_{H}$ represents the number of infected humans. $N_{M}$ denotes the total population number where the subscripts H and M stand for the host and mosquito, respectively. $\alpha$ stands for the random rate of dengue seeding into the local area, $U$is the dissemination rate and is constant over an outbreak, $\mu_{b}(t)$ is the mosquito birth rate at time t, $\mu_{d}$ is the mosquito death rate and is constant over an outbreak, $\tau(t)$ denotes the transmission rate, dictating the speed at which susceptible individuals become infectious. $\beta_{0}$ is the basic contact rate between humans and mosquitoes, $\beta(t)$ is the contact rate between humans and mosquitoes at time t, $D$ is the mean infectious period for human.

This model establishes a standard susceptible-infected-recovered (SIR) epidemiological structure within mosquito and human populations, respectively, with humans connected to mosquitoes by mosquito bites. All compartments in the model were completely mixed. During each outbreak, human population size was assumed to be constant, and no births or deaths were simulated, while mosquito population size varied with seasonal mosquito birth rate and constant mortality driven by ambient temperature [4]. In addition, dengue can be transmitted vertically by infected male mosquitoes mating with females [5]. Therefore, a constant parameter is introduced to account for the probability of vertical transmission of infected mosquitoes. A random infection rate parameter was also introduced into the model to represent infected mosquitoes randomly in the mosquito population or was introduced from locations outside the study area.

1. **Data assimilation methods**

In recent years, data assimilation methods have been applied in infectious disease prediction [6-10]. This framework consists of observations, a dynamic model that simulates the dynamics of the underlying system, and a data assimilation method that integrates information from the system observations into the model. The ensemble adjusted Kalman filter (EAKF) [11] has performed well in existing studies of infectious disease prediction. The EAKF could estimate the real state given the observations and state simulation. This algorithm assumes that the state vector and observation at time t are $x_{t}$ and $Y_{t}$, respectively. The prior distribution of the system state at time t, $p(x_{t}|Y_{1:t-1})$ can be updated to a posterior distribution $p(x_{t}|Y_{1:t})$ using Bayes' rule

$$p(x_{t}|Y_{1:t})=p(x_{t}|Y_{1:t-1})p(Y_{t}|x_{t})/p(Y_{t}|Y_{1:t-1})$$

where the second part of the numerator is the likelihood probability and the denominator is proven to be constant using Law of Total Probability. Therefore, the posterior probability is positively correlated with the prior probability.

$$p(x_{t}|Y_{1:t})\propto p(x_{t}|Y_{1:t-1})p(Y_{t}|x_{t})$$

Assuming that both likelihood probability and prior probability were subject to Gaussian distribution, then this formula can be seen as a product of two Gaussian distribution probability density functions, resulting in a Gaussian distribution. According to the formula of the product of Gaussian distribution:

$$\mu_{fg}=\frac{\mu_{f}\sigma_{g}^{2}+\mu_{g}\sigma_{f}^{2}}{\sigma_{f}^{2}+\sigma_{g}^{2}}$$

$$\sigma_{fg}^{2}=\frac{\sigma_{f}^{2}\sigma_{f}^{2}}{\sigma_{f}^{2}+\sigma_{f}^{2}}$$

The mean and variance of the obtained Gaussian distribution are

$$\sum u=[(\sum p)^{-1}+\mathbf{H}^{T}\mathbf{R}^{-1}\mathbf{H}]^{-1}$$

$$\bar{z}^{u}=\sum u[(\sum p)^{-1}\bar{z}^{p}+\mathbf{H}^{T}\mathbf{R}^{-1}\mathbf{y}^{o}]$$

EAKF optimized the set of model-simulated state variables to the real state. EAKF also adjusts unobserved state variables and parameters based on their covariability with observed state variables. In Kalman filter, the relationship between these variables is assumed to be linear. Thus, the adjustment of unobserved state variables and parameters is linearly related to the adjustment of observed values through their covariance, which is calculated directly from the ensemble.

It's worth mentioning that the Kalman filters assume that both the prior distribution and likelihood are Gaussian, thus the distributions can be fully parameterized by the first two moments (mean and covariance). Instead of using random perturbations with stochasticity, as in other forms of the Kalman filter, the EAKF adjusts the ensemble members deterministically, so that the covariance of the prior distribution is preserved in posterior. Moreover, higher moment structure is also retained during the update.

**References**

1. Sugihara G, May R, Ye H, Hsieh CH, Deyle E, Fogarty M, et al. Detecting causality in complex ecosystems. Science (New York, NY). 2012;338(6106):496-500. Epub 2012/09/22. doi: 10.1126/science.1227079. PubMed PMID: 22997134.

2. Nova N, Deyle ER, Shocket MS, MacDonald AJ, Childs ML, Rypdal M, et al. Susceptible host availability modulates climate effects on dengue dynamics. Ecology letters. 2021;24(3):415-25. Epub 2020/12/11. doi: 10.1111/ele.13652. PubMed PMID: 33300663; PubMed Central PMCID: PMCPMC7880875.

3. Chen Y, Liu T, Pei S, Yu X, Zeng Q, Wu H, et al. Ensemble Forecasts of Seasonal Dengue Epidemics. medRxiv; 2021.

4. Brady OJ, Golding N, Pigott DM, Kraemer MU, Messina JP, Reiner RC, Jr., et al. Global temperature constraints on Aedes aegypti and Ae. albopictus persistence and competence for dengue virus transmission. Parasites & vectors. 2014;7:338. Epub 2014/07/24. doi: 10.1186/1756-3305-7-338. PubMed PMID: 25052008; PubMed Central PMCID: PMCPMC4148136.

5. Bosio CF, Thomas RE, Grimstad PR, Rai KS. Variation in the efficiency of vertical transmission of dengue-1 virus by strains of Aedes albopictus (Diptera: Culicidae). Journal of medical entomology. 1992;29(6):985-9. Epub 1992/11/01. doi: 10.1093/jmedent/29.6.985. PubMed PMID: 1460640.

6. Reich NG, McGowan CJ, Yamana TK, Tushar A, Ray EL, Osthus D, et al. Accuracy of real-time multi-model ensemble forecasts for seasonal influenza in the U.S. PLoS computational biology. 2019;15(11):e1007486. Epub 2019/11/23. doi: 10.1371/journal.pcbi.1007486. PubMed PMID: 31756193; PubMed Central PMCID: PMCPMC6897420 following competing interests: JS and Columbia University disclose partial ownership of SK Analytics.

7. Yang W, Karspeck A, Shaman J. Comparison of filtering methods for the modeling and retrospective forecasting of influenza epidemics. PLoS computational biology. 2014;10(4):e1003583. Epub 2014/04/26. doi: 10.1371/journal.pcbi.1003583. PubMed PMID: 24762780; PubMed Central PMCID: PMCPMC3998879.

8. DeFelice NB, Little E, Campbell SR, Shaman J. Ensemble forecast of human West Nile virus cases and mosquito infection rates. Nature communications. 2017;8:14592. Epub 2017/02/25. doi: 10.1038/ncomms14592. PubMed PMID: 28233783; PubMed Central PMCID: PMCPMC5333106 no competing financial interests.

9. Yang W, Cowling BJ, Lau EH, Shaman J. Forecasting Influenza Epidemics in Hong Kong. PLoS computational biology. 2015;11(7):e1004383. Epub 2015/08/01. doi: 10.1371/journal.pcbi.1004383. PubMed PMID: 26226185; PubMed Central PMCID: PMCPMC4520691 following competing interests: BJC has received research funding from MedImmune Inc. and Sanofi Pasteur, and consults for Crucell NV. JS discloses consulting for JWT and Axon Advisors, as well as partial ownership of SK Analytics. The authors report no other potential conflicts of interest. This does not alter our adherence to all PLOS policies on sharing data and materials.

10. Shaman J, Karspeck A, Yang W, Tamerius J, Lipsitch M. Real-time influenza forecasts during the 2012-2013 season. Nature communications. 2013;4:2837. Epub 2013/12/05. doi: 10.1038/ncomms3837. PubMed PMID: 24302074; PubMed Central PMCID: PMCPMC3873365.

11. Anderson JL. An Ensemble Adjustment Kalman Filter for Data Assimilation %J Monthly Weather Review. 2001;129(12):2884-903. doi: 10.1175/1520-0493(2001)129<2884:Aeakff>2.0.Co;2.
