# Supplementary figures and images for: Dengue transmission dynamics prediction by combining metapopulation networks and Kalman filter algorithm
Source: PLoS Negl Trop Dis. 2023 Jun 7;17(6):e0011418. doi: 10.1371/journal.pntd.0011418 (PMC10281582; doi:10.1371/journal.pntd.0011418)

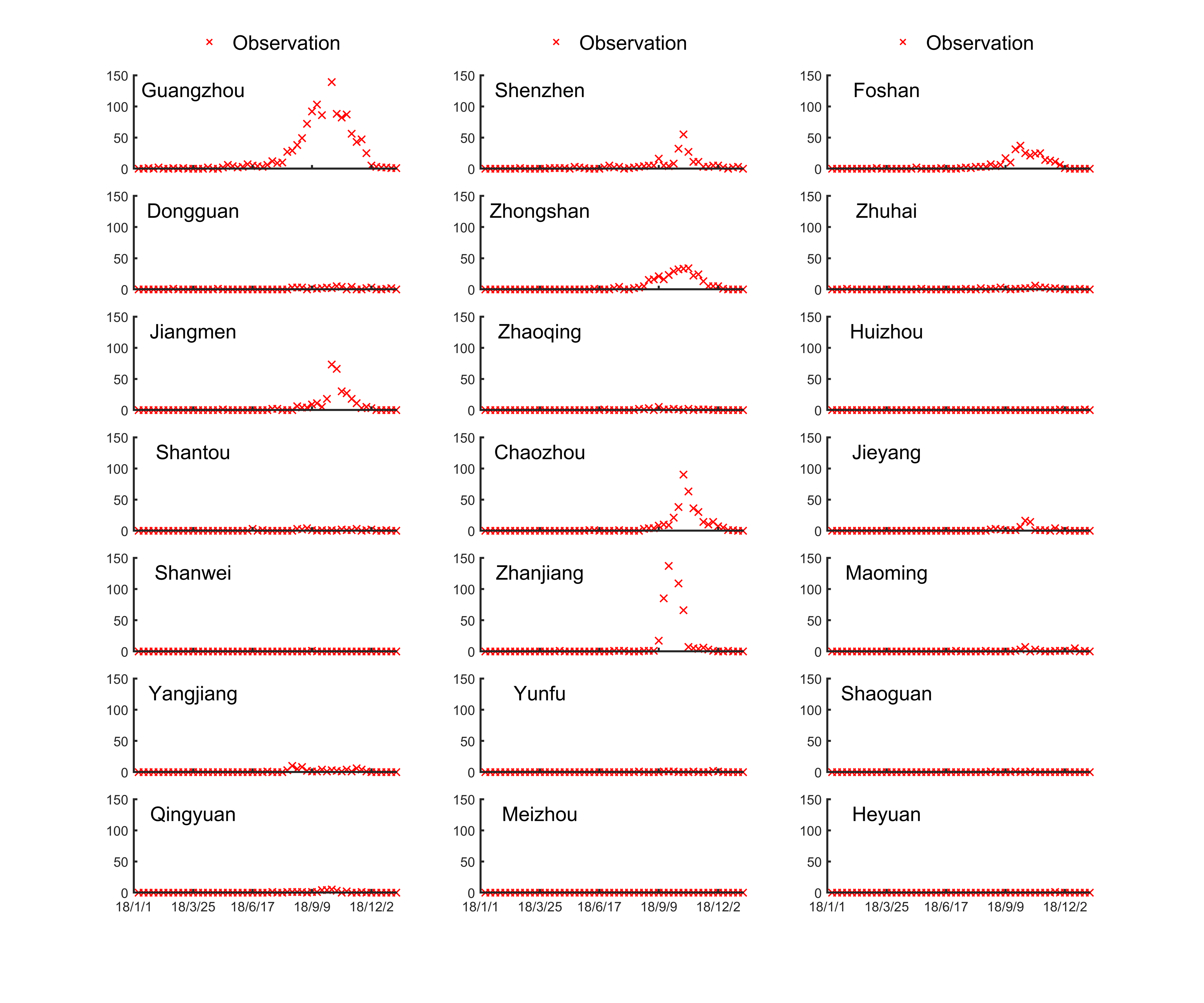

Supplement: S1 Fig — (TIF) [file pntd.0011418.s002.tif]

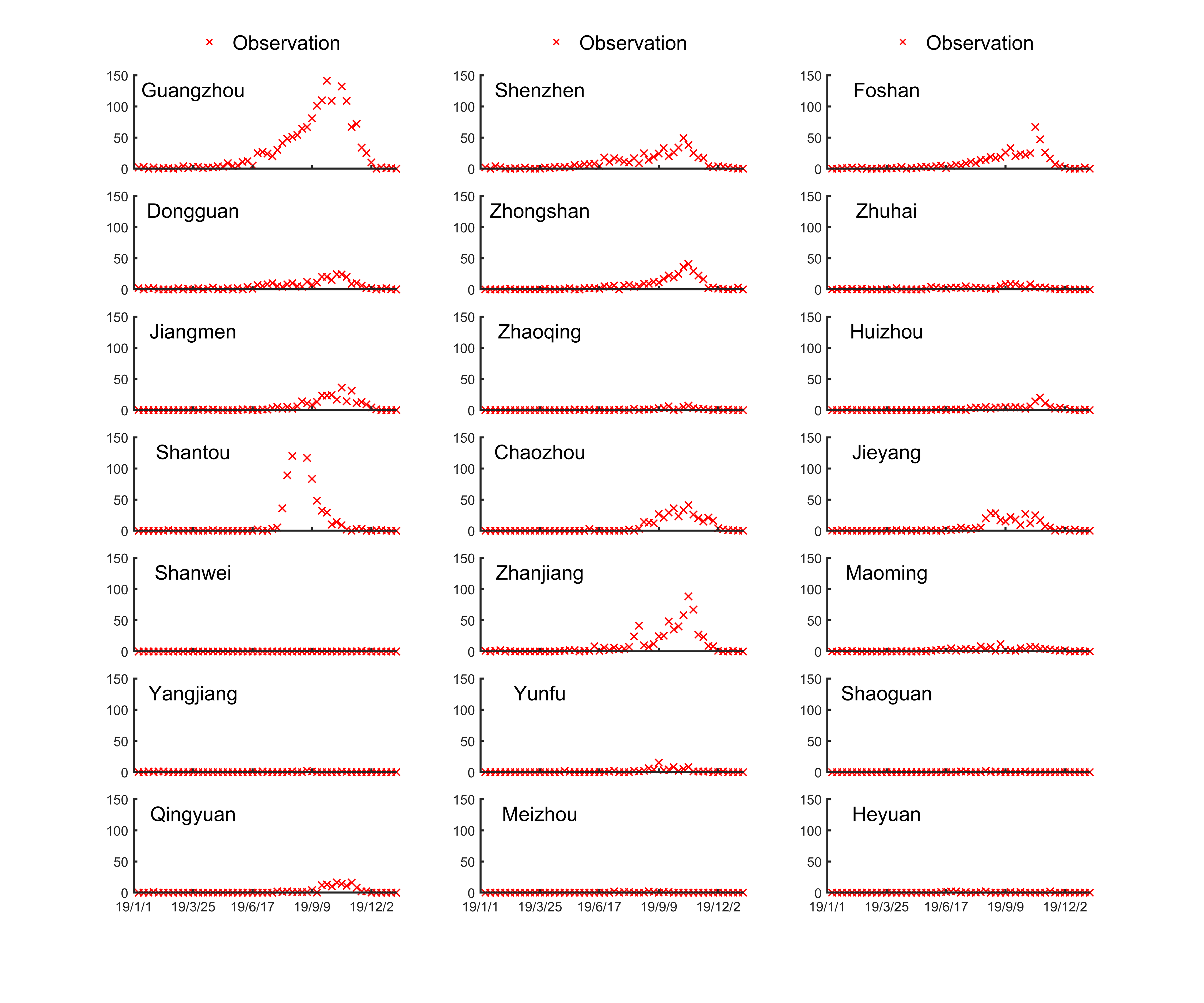

Supplement: S2 Fig — (TIF) [file pntd.0011418.s003.tif]

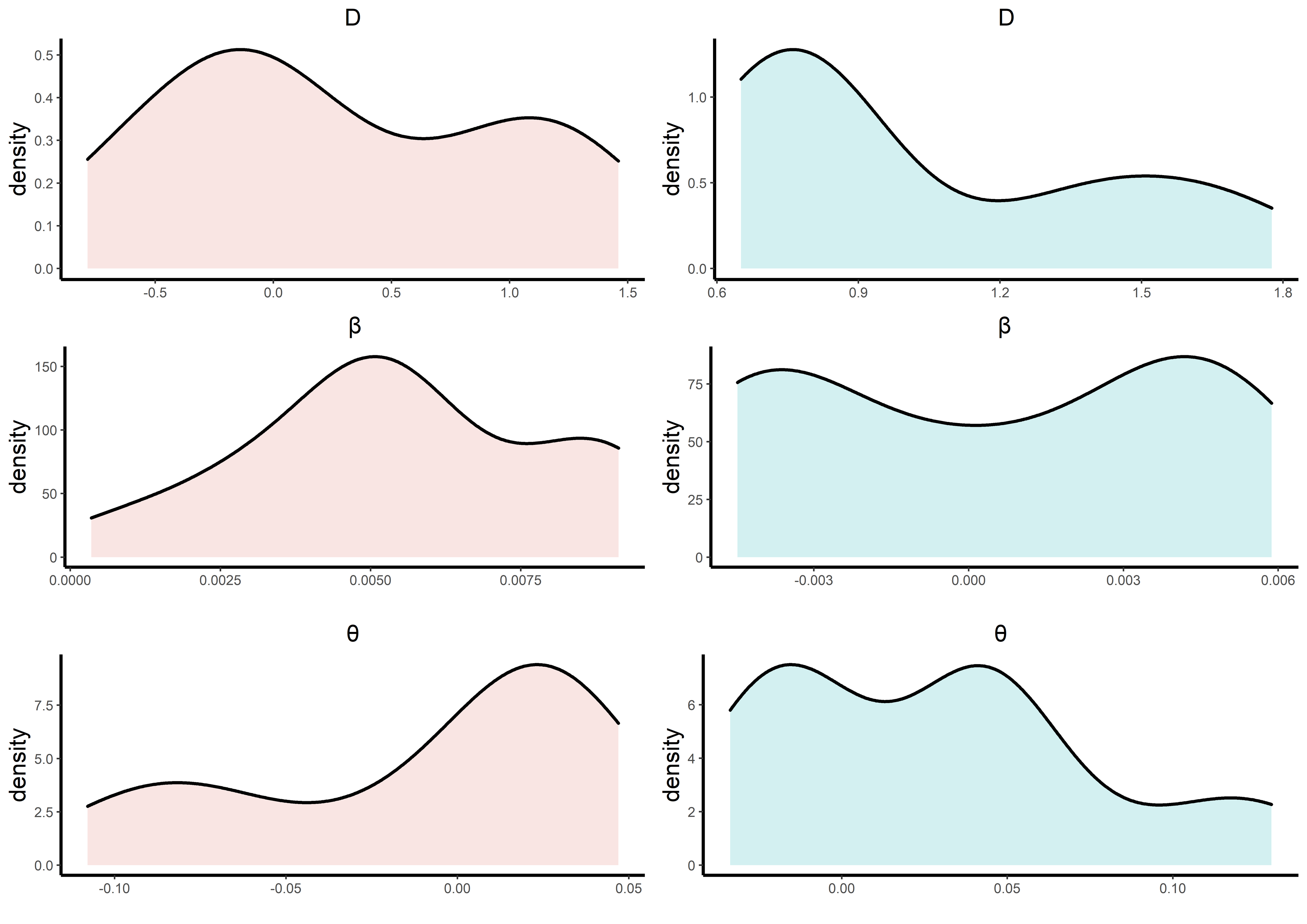

Supplement: S3 Fig — For θ = 0.05 (left) and θ = 0.25 (right), the relative error distribution of D, β, and θ in the 21st week was deduced by 300 independent estimations using the metapopulation model. Each estimate is the mean of the randomly selected 300 member sets. (TIF) [file pntd.0011418.s004.tif]

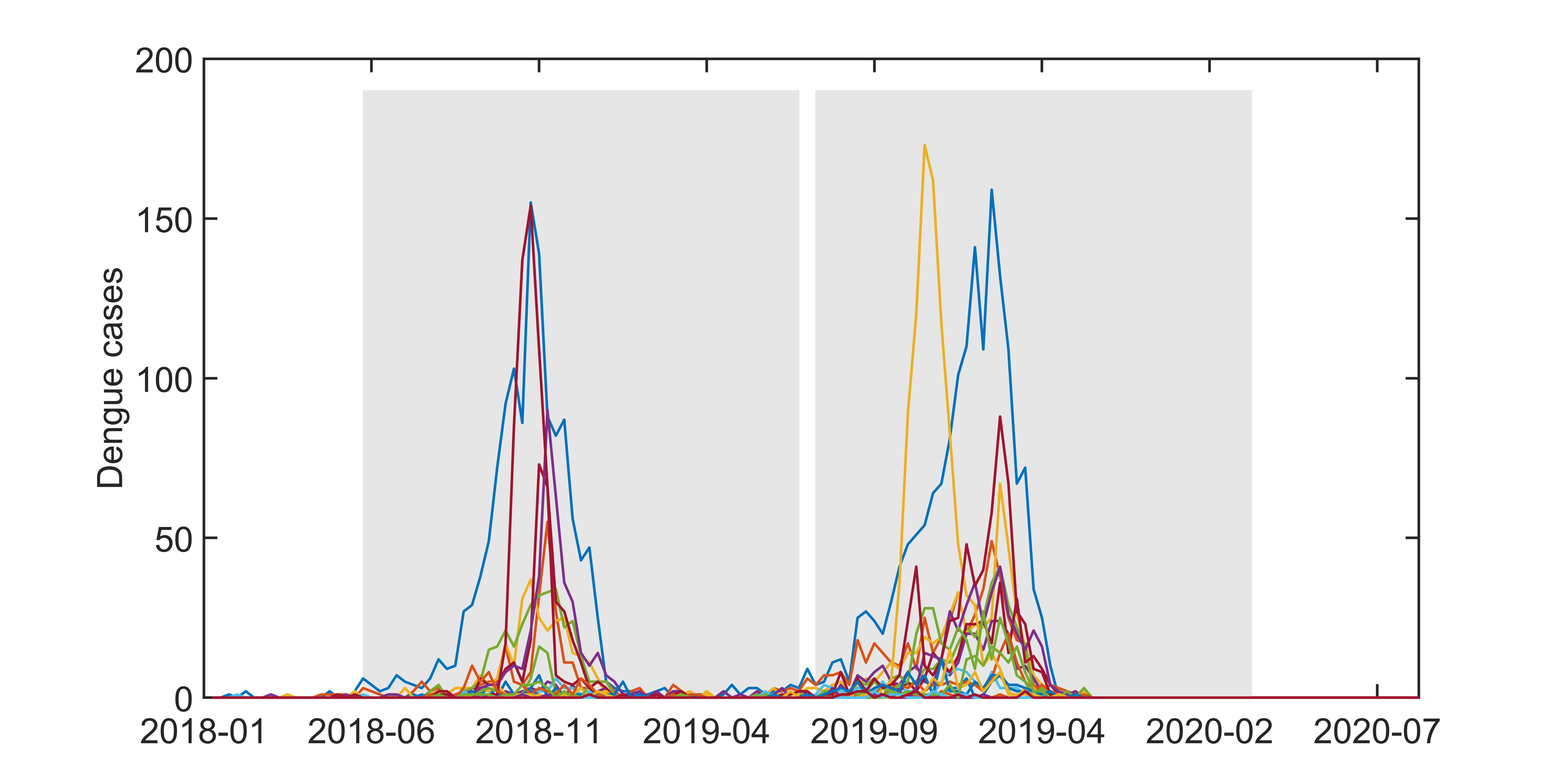

Supplement: S4 Fig — The retrospective forecast season is marked by grey areas. (TIF) [file pntd.0011418.s005.tif]

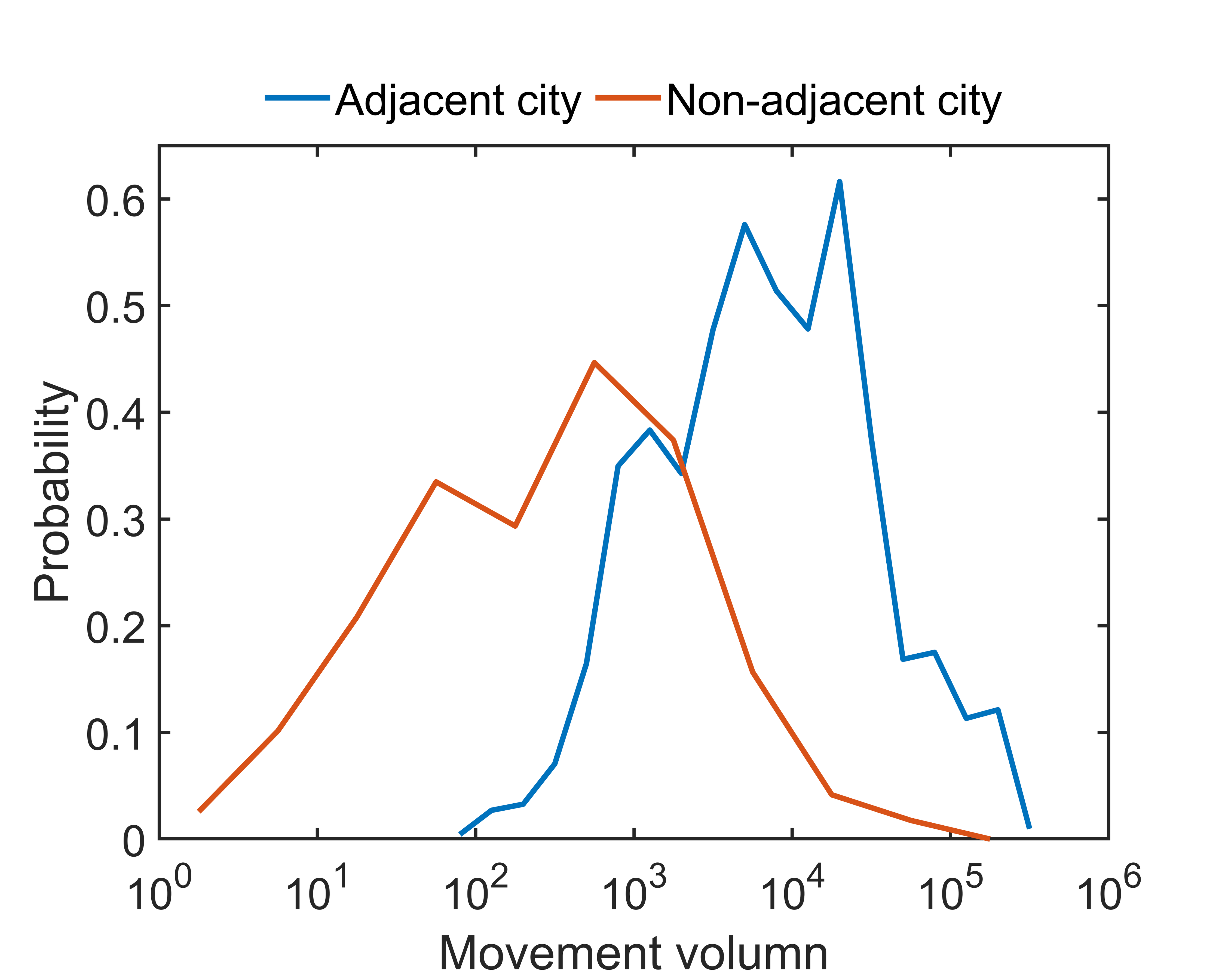

Supplement: S5 Fig — (TIF) [file pntd.0011418.s006.tif]

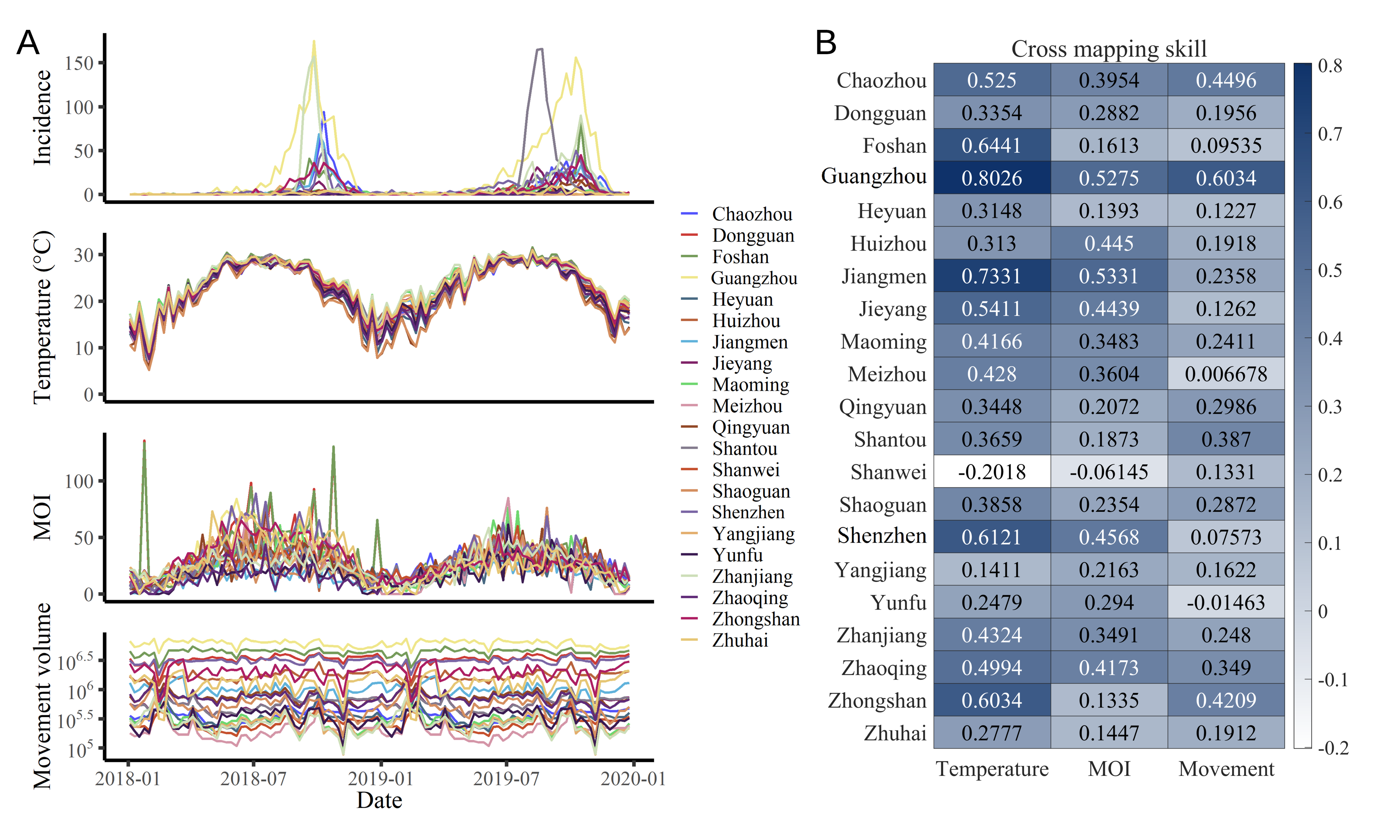

Supplement: S6 Fig — (A) weekly observations of dengue fever, temperature, Mosquito Oviposition Index (MOI), and population movements for each prefecture-level city in Guangdong Province from 2018 to 2020. (B) Climate, population movement data and mosquito vector MOI index drive dengue incidence, and Cross mapping skills were calculated for each city in Guangdong. (TIF) [file pntd.0011418.s007.tif]

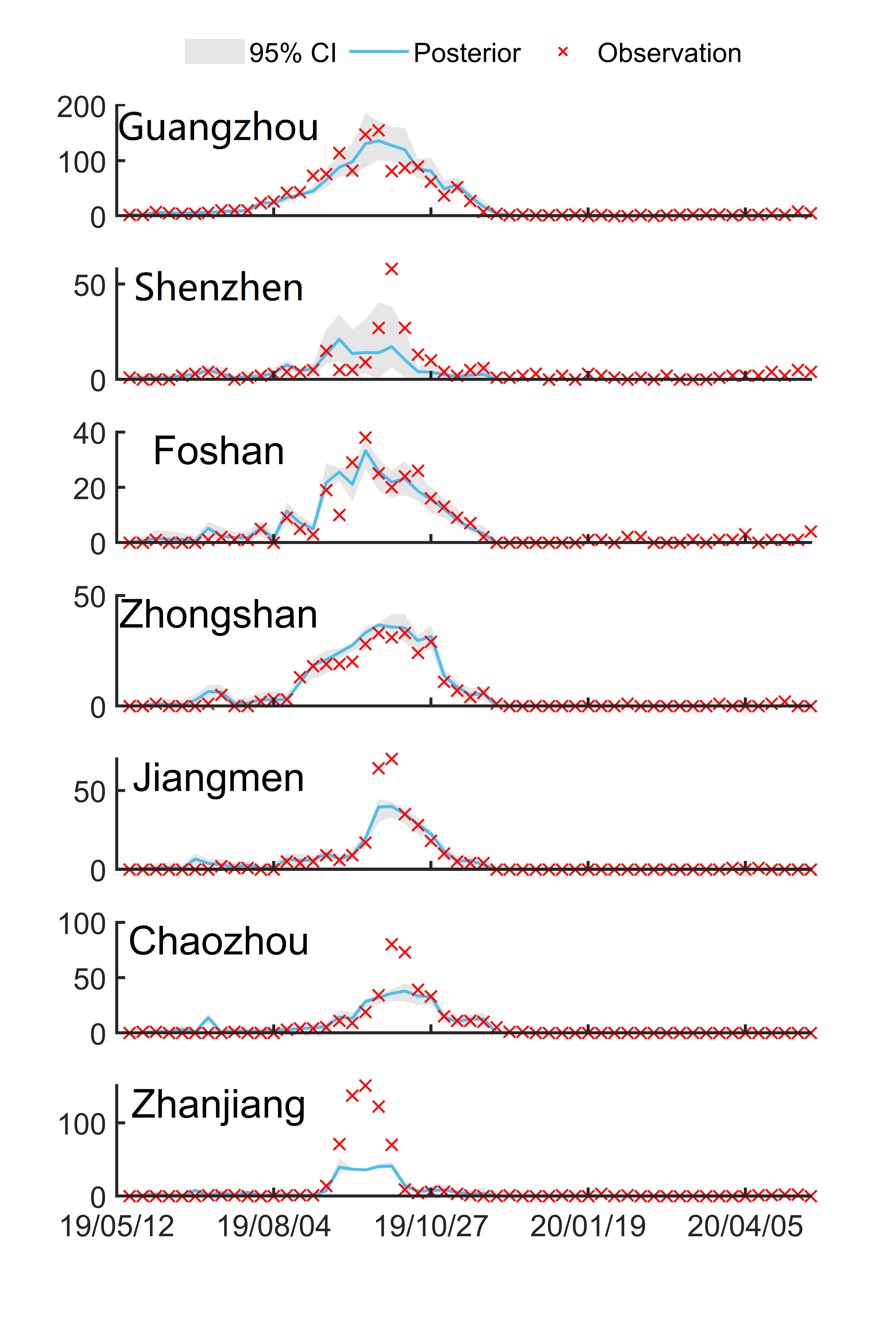

Supplement: S7 Fig — Weekly dengue fever cases (cross symbols) for each prefecture-level city. The solid lines and shaded areas are the posterior mean and 95% credible intervals (CI), respectively, of the metapopulation network-EAKF fit. (TIF) [file pntd.0011418.s008.tif]

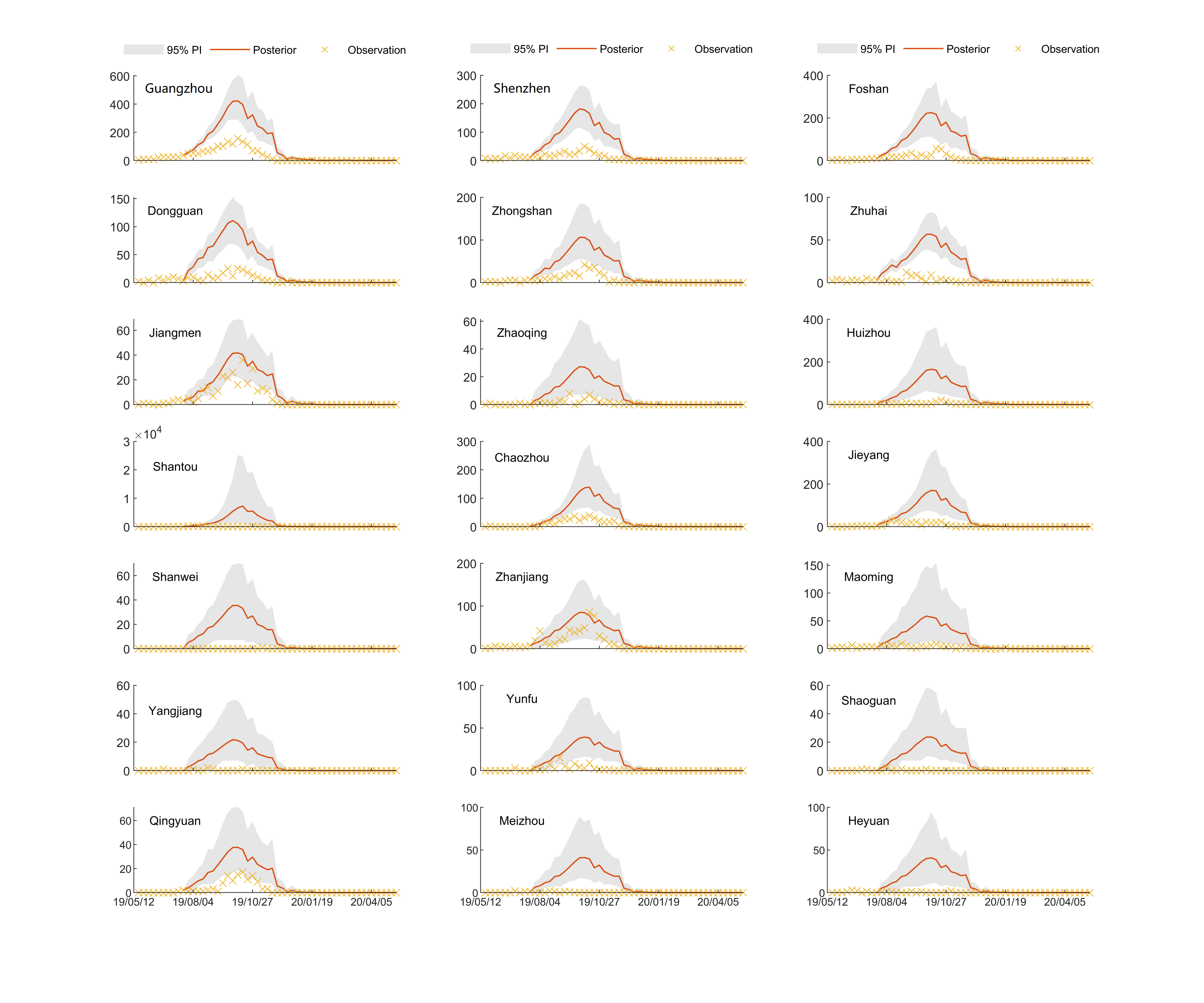

Supplement: S8 Fig — The metapopulation model with 300 ensemble members is used. The yellow cross symbols indicate weekly observations. The solid red curves are average forecast trajectories, and the grey areas represent 95% prediction intervals. (TIF) [file pntd.0011418.s009.tif]

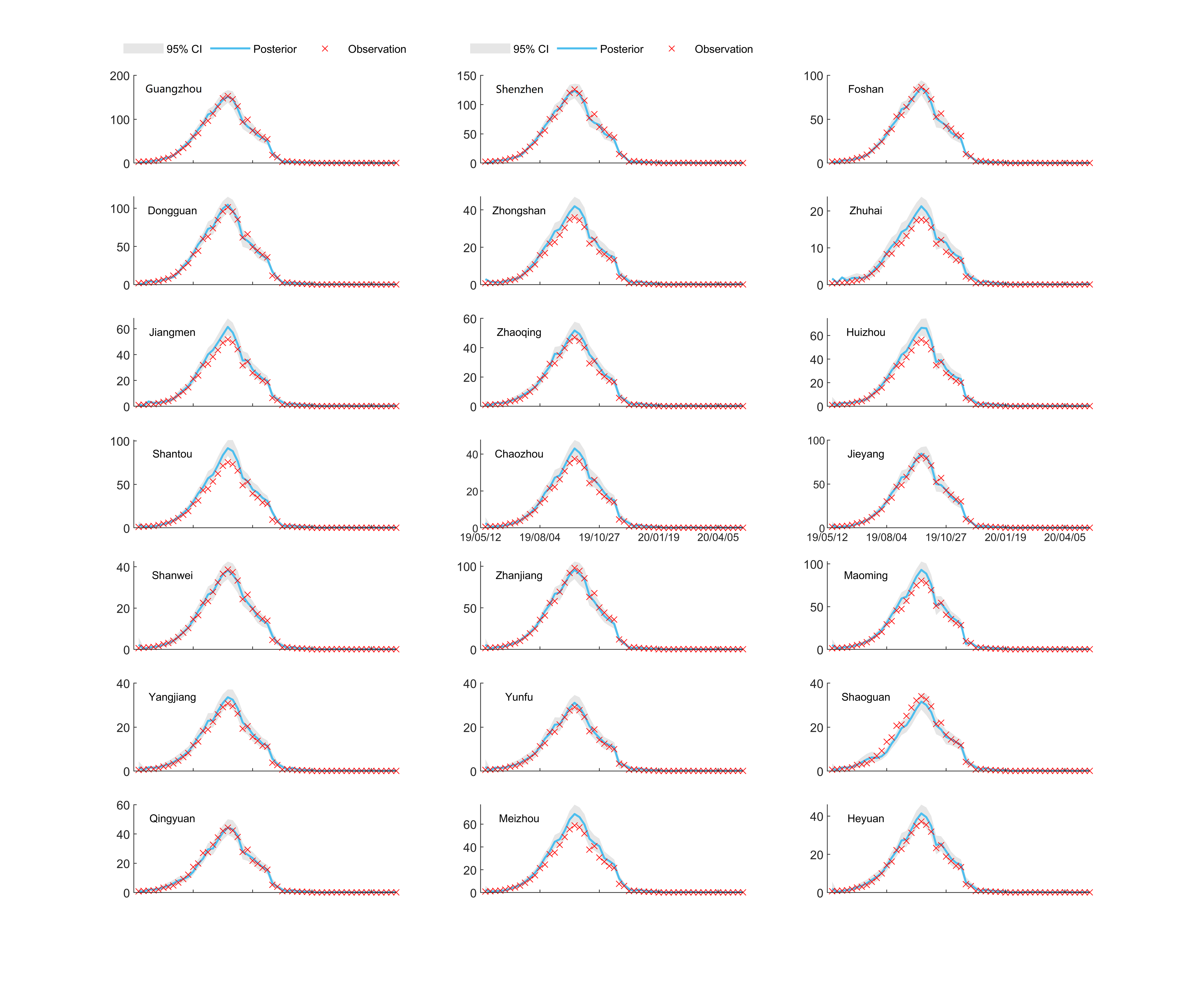

Supplement: S9 Fig — Simulations were obtained under the same initial conditions, and the random movement parameter is set as θ = 0.05. Posterior mean estimates of weekly observation of new cases in metapopulation simulations are displayed for 21 cities separately. The solid line and shadow area are the posterior mean and 95% confidence interval (CI) of the metapopulation network-EAKF fitting, respectively. The red cross symbols indicate the synthetic observations used for data assimilation. (TIF) [file pntd.0011418.s010.tif]

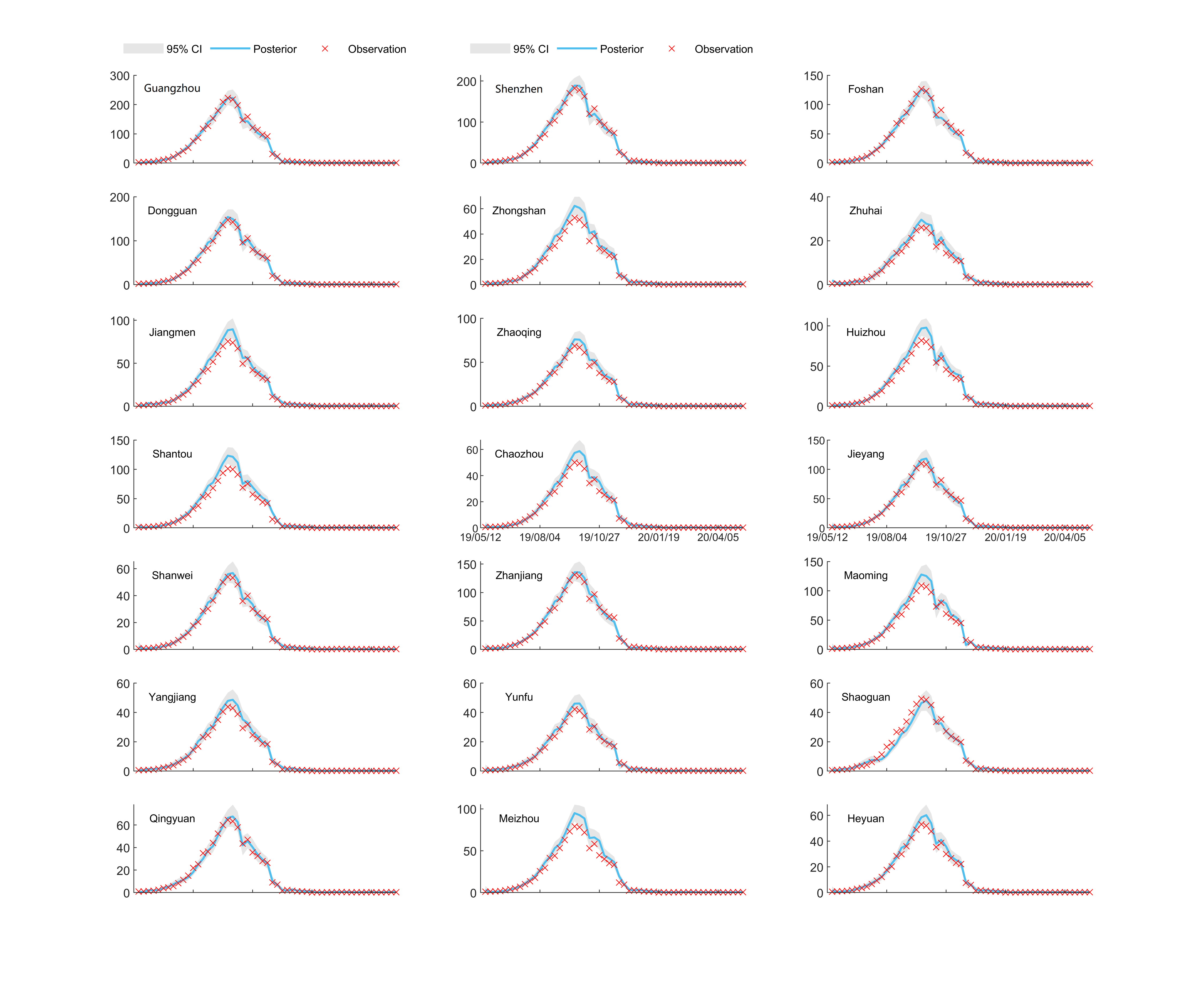

Supplement: S10 Fig — Simulations were obtained under the same initial conditions, and the random movement parameter is set as θ = 0.25. Posterior mean estimates of weekly observation of new cases in metapopulation simulations are displayed for 21 cities separately. The solid line and shadow area are the posterior mean and 95% confidence interval (CI) of the metapopulation network-EAKF fitting, respectively. The red cross symbols indicate the synthetic observations used for data assimilation. (TIF) [file pntd.0011418.s011.tif]

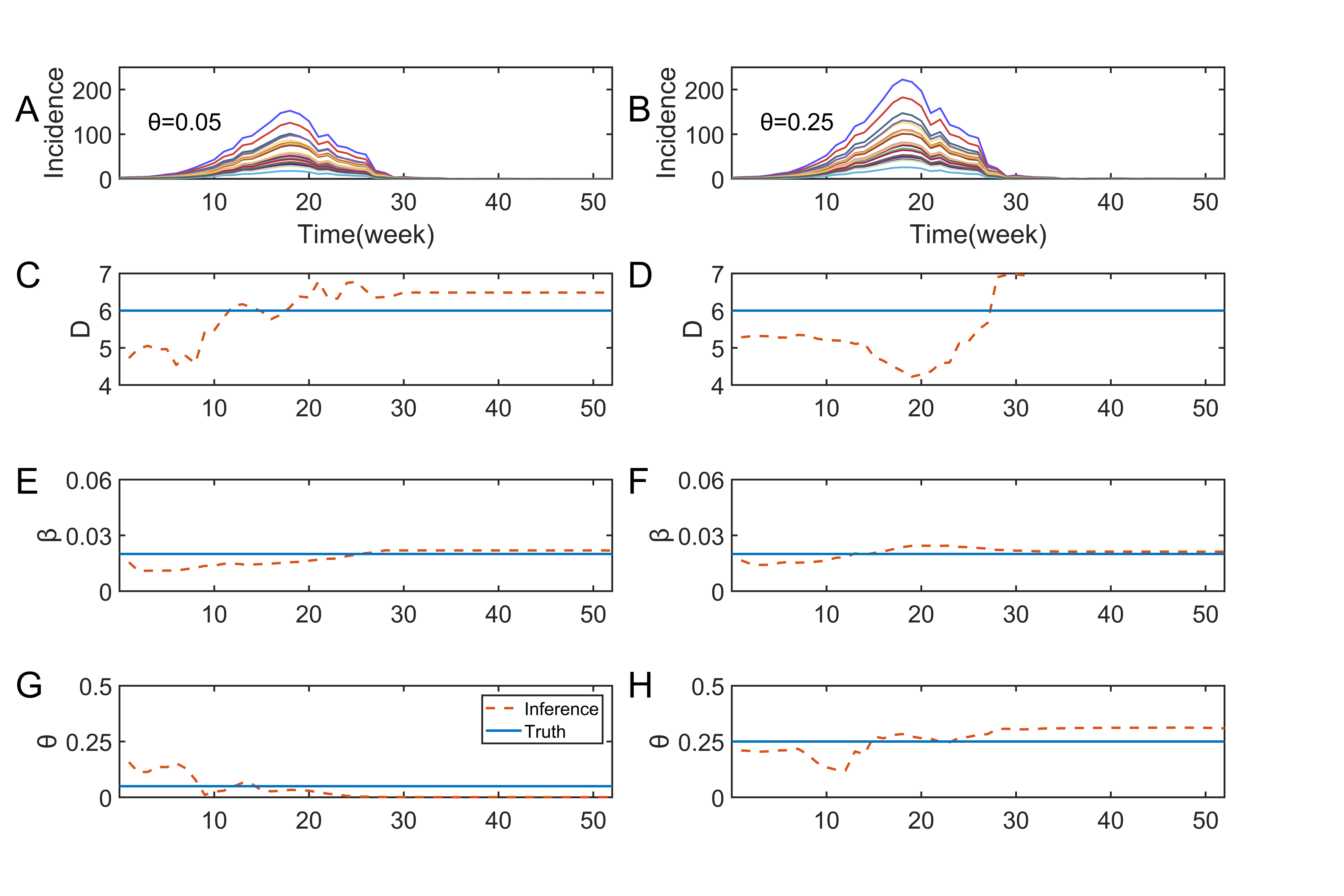

Supplement: S11 Fig — (A and B) The metapopulation model generates the number of new infections per week in 21 cities. Except (A) θ = 0.05 and (B) θ = 0.25, both simulations were obtained under the same initial conditions. Different colors distinguish the epidemic curves of new cases in different cities. (C θ = 0.05; D θ = 0.25) Inference of the parameter D (infection duration) by the metapopulation network-EAKF system. The real blue line represents the real parameters used in the simulated epidemics, and the red dotted line represents the posterior mean in the data assimilation process. (E θ = 0.05; F θ = 0.25) The parameter β (contact rate) in the metapopulation system is inferred. (G θ = 0.05; H θ = 0.25) Inference of the parameter θ (random movement ratio) in the metapopulation model. (TIF) [file pntd.0011418.s012.tif]

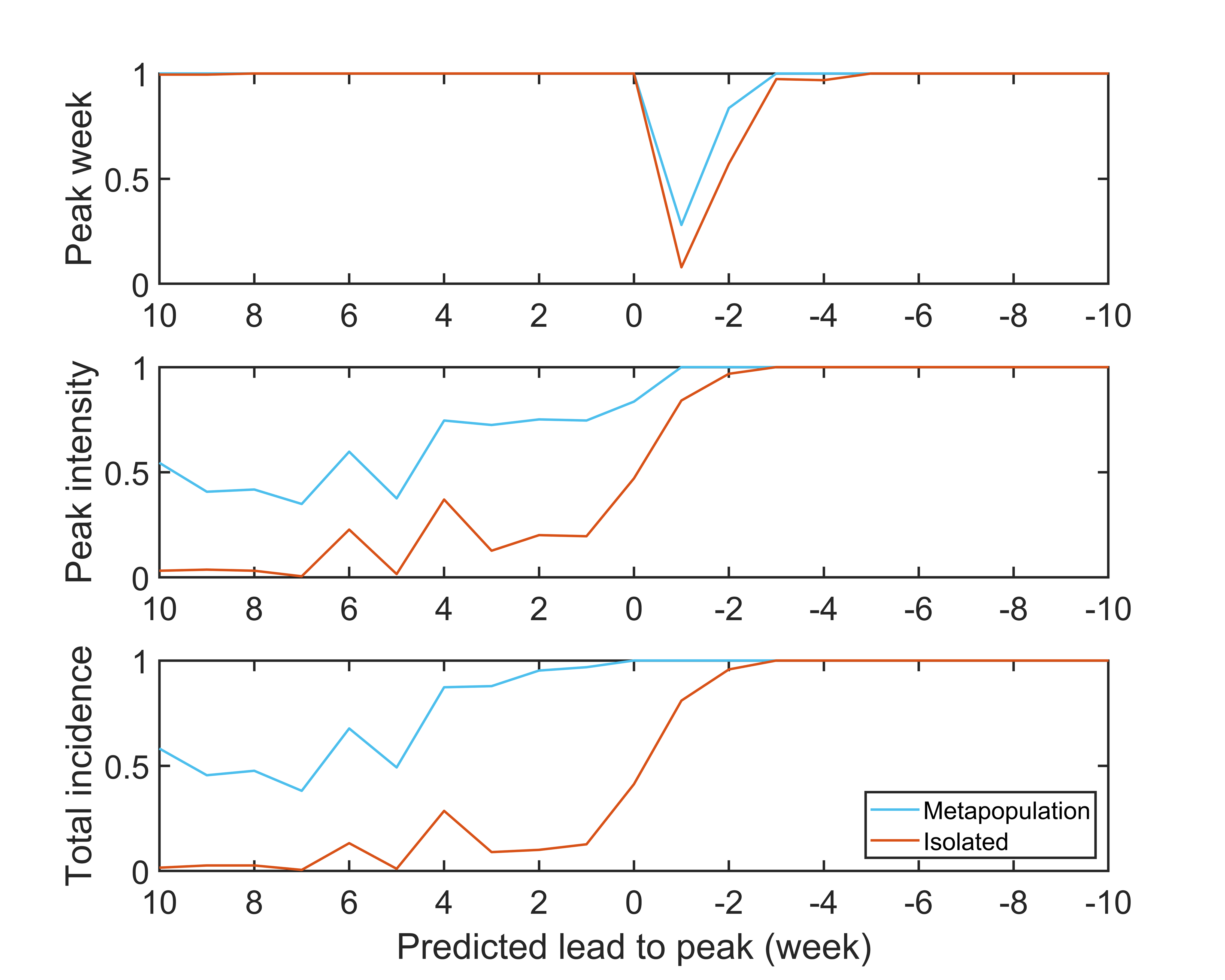

Supplement: S12 Fig — Based on population movement, ambient temperature, and Mosquito Oviposition Index data from 21 cities in Guangdong, 10 synthetic outbreaks were generated using an ensemble population model. For each synthetic outbreak, weekly forecasts were made using 300-member ensembles. (TIF) [file pntd.0011418.s013.tif]

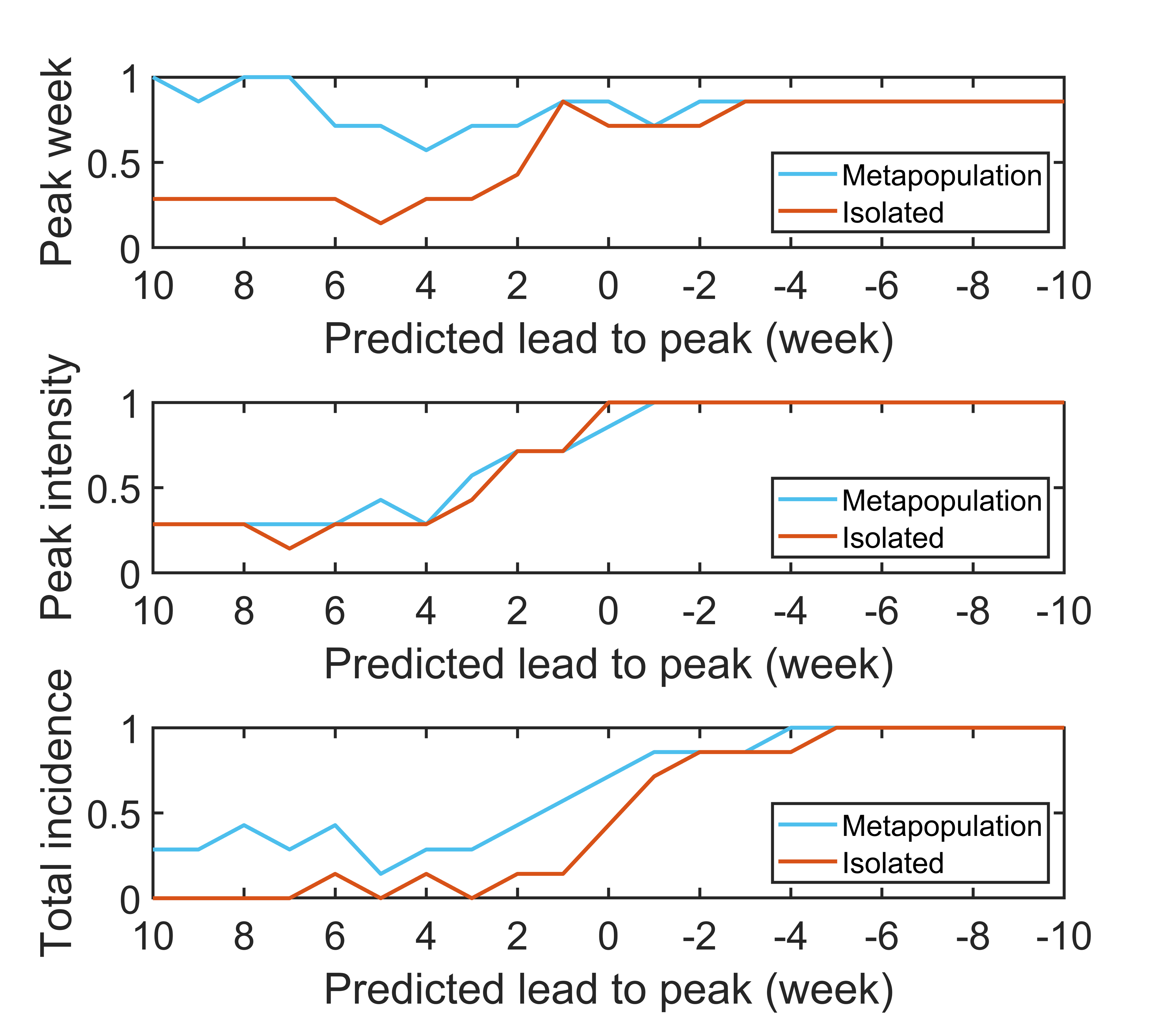

Supplement: S13 Fig — (TIF) [file pntd.0011418.s014.tif]

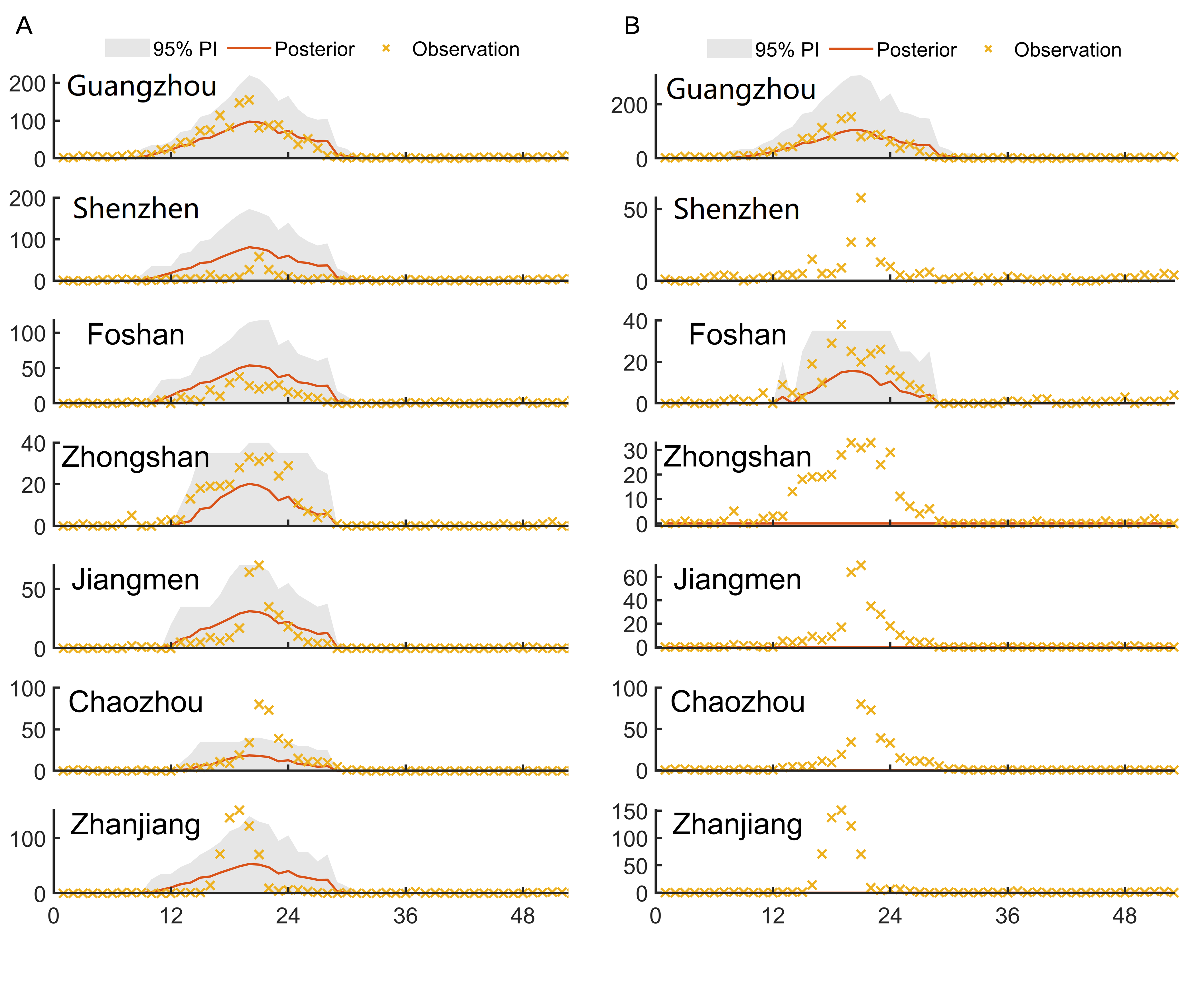

Supplement: S14 Fig — Two forecast systems predicted the integrated epidemic curve of seven cities in week 10. Research provides prediction methods using metapopulation model (A) and isolated model (B) prediction systems. Three hundred ensemble members are used in the forecast. 95% prediction intervals (PIs) are reported. The metapopulation forecast system well predicts the outbreak curve. Although the Isolated model captured seasonal targets such as peak week in a few locations, the overall epidemic curve is underestimated by isolated prediction. (TIF) [file pntd.0011418.s015.tif]
