# Supplementary material for: Dengue transmission dynamics prediction by combining metapopulation networks and Kalman filter algorithm
Source: PLoS Negl Trop Dis. 2023 Jun 7;17(6):e0011418. doi: 10.1371/journal.pntd.0011418 (PMC10281582; doi:10.1371/journal.pntd.0011418)
Supplement: S1 Table — (DOCX) [file pntd.0011418.s016.docx]

**S1 Table**.  **The number of cases predicted by metapopulation and isolated model before peak week.**

| Prediction lead to peak | 10 week | 9 week | 8 week | 7 week | 6 week | 5 week | 4 week | 3 week | 2 week | 1 week | 0 week |
| --- | --- | --- | --- | --- | --- | --- | --- | --- | --- | --- | --- |
| Metapopulation forecast | 330 | 210 | 204 | 134 | 49 | 24 | 31 | 22 | 22 | 17 | 14 |
| Isolated forecast | 3 | 5 | 8 | 13 | 19 | 11 | 14 | 7 | 11 | 13 | 12 |
