# Supplementary material for: Dengue transmission dynamics prediction by combining metapopulation networks and Kalman filter algorithm
Source: PLoS Negl Trop Dis. 2023 Jun 7;17(6):e0011418. doi: 10.1371/journal.pntd.0011418 (PMC10281582; doi:10.1371/journal.pntd.0011418)
Supplement: Attachment — Submitted filename: Response to Reviewers.doc [file pntd.0011418.s018.doc]

**Manuscript Number:** PNTD-D-22-00643

**Title:** Dengue transmission dynamics prediction by combining metapopulation networks and Kalman filter algorithm

**Journal:** PLOS Neglected Tropical Diseases

Dear Professor Eric HY Lau and Robert Reiner,

Thank you very much for giving us the opportunity to revise our manuscript. We strongly endorse the three reviewers' suggestions, which have greatly improved our manuscript. Every change has been outlined and the responses to the comments have been made point by point. We have also expressed our sincere appreciation of your help in the Acknowledgment section of the manuscript. Our responses to the comments have been given in the next page.

In this round of revision, we have submitted the files as follows:

1. A cover letter (uploaded as a **"Cover Letter" file**) was uploaded.

2. A letter (uploaded as a **"Response to Reviewers" file**) containing a detailed list of our responses to the review comments and a description of the changes we have made was uploaded.

3. A marked-up copy with tracked changes denoting where the text has been changed (uploaded as a **"Revised Manuscript with Changes Marked" file**) was uploaded.

4. A clean revised manuscript (uploaded as a **"Manuscript" file**) was submitted.

5. All the figures were uploaded separately, and a supplementary material file (uploaded as a **"Supporting Information" file**) was also uploaded.

Yours sincerely,

Pi Guo

Department of Preventive Medicine, Shantou University Medical College

Email: pguo@stu.edu.cn

**To Editor:**

**1) Comment:** “the authors are expected to address all the criticisms by all Reviewers. In particular, please describe the novel contribution of your model, and implications in terms of early warning or other interventions (Reviewers #1 and #2), further elaborate your model and meta-population structure (Reviewers #1 and #3), and clarify whether change in human mobility due to the COVID-19 pandemic has been considered or incorporated (Reviewers #2 and #3)”.

**Response:** Dear editor, thank you for your encouragement. First, we feel sorry that the objectives of the study and its novel contribution are not well described. In fact, our research was methodologically innovative. We have revised **the Introduction part** and **the Discussion part** according to the comment #9 from Reviewer #2. Besides, you have also pointed out the implications in terms of early warning or other interventions, we have added statements in **the Abstract part, and the last paragraph of the Discussion part** based on the comment #3 from Reviewer #1. Further, we have elaborated the model to Reviewer #1, and added necessary statements in this revised version of manuscript. To elaborate the meta-population structure, we have revised the manuscript by adding necessary theoretical figure and revised relevant statement according to the comment #15 from Reviewer #2. Please check it in **the updated Figure 2 and the Methods part**. In addition, we have shown the retrospective forecast season is marked by grey areas in Fig. S4. The 2019-2020 retrospective forecasting season in our study ends in February 2020. At this time, the outbreak of COVID-19 has only occurred in Wuhan, Hubei province, and has not yet spread to Guangdong province. Therefore, we believe that the results of this study would probably not have been affected by the epidemic. We have carefully revised the manuscript according to your suggestions. We hope the revisions will make you feel appropriate and finally be approved by you.

**2) Comment:** "Figure Files: While revising your submission, please upload your figure files to the Preflight Analysis and Conversion Engine (PACE) digital diagnostic tool, https://pacev2.apexcovantage.com. PACE helps ensure that figures meet PLOS requirements. To use PACE, you must first register as a user. Then, login and navigate to the UPLOAD tab, where you will find detailed instructions on how to use the tool. If you encounter any issues or have any questions when using PACE, please email us at figures@plos.org."

**Response:** Dear editor, thank you for your suggestion. We have uploaded the figure files to the PACE digital diagnostic tool and checked that figures meet PLOS requirements.

**3) Comment:** " Data Requirements: Please note that, as a condition of publication, PLOS' data policy requires that you make available all data used to draw the conclusions outlined in your manuscript. Data must be deposited in an appropriate repository, included within the body of the manuscript, or uploaded as supporting information. This includes all numerical values that were used to generate graphs, histograms etc. For an example see here: http://www.plosbiology.org/article/info%3Adoi%2F10.1371%2Fjournal.pbio.1001908#s5."

**Response:** Dear editor, thank you very much. The dengue surveillance data used in this study cannot be publicly available by uploading as supporting information due to the current confidentiality agreement on infectious disease surveillance data in China. In fact, the data are deposited in an appropriate repository. However, people can request access to the data from the National Population Health Science Data Sharing Platform. Therefore, for data availability, we have added the statement that “To protect the privacy of study participants, primary data are not publicly available. To access these data and/or to seek permission for its use, please contact the Data-center of China Public Health Science (https://www.phsciencedata.cn/Share/index.jsp) or email data@chinacdc.cn” in the manuscript submission system. Please check it. Thank you for your understanding.

**To Reviewer #1:**

Dear professor, we really thank you for your encouragement that "the manuscript entitled ‘Dengue transmission dynamics prediction by combining metapopulation networks and Kalman filter algorithm’ applied mathematical model incorporated with EAKF approach to simulate dengue transmission in Guangdong. Overall, the models and results are sound". We really appreciate the insightful comments that "some detailed in the modeling approaches should be clarified to improve the manuscript". We really cherish this opportunity and have carefully revised the manuscript according to your comments. We hope our revisions have made it more understandable for potential readers. Our responses to the comments have been arranged one by one in the following section.

**1) Comment:** "The way to avoid zero-inflated issue is a little confusing. The author just shifted the initial time point to the 19th week rather than the first week of the year. However, the weekly data still contains many zeros. Could you explain more about why the changes can deal with the zero-inflated issue?"

**Response:** Dear Professor, thank you very much for this comment. We feel sorry about the poor language in the previous version of manuscript that makes you confused. Shifting the initial time point to the 19th week rather than the first week of the year was not to deal with the zero-inflated issue. We'd like to take this opportunity to explain to you that we have dealt with the zero inflation issue of the data by using the EAKF algorithm, which has been shown to process dengue zero inflation data in our previous studies (please refer to: Chen Y, et.al. An ensemble forecast system for tracking dynamics of dengue outbreaks and its validation in China. PLoS Comput Biol. 2022). Besides, the effect of filtering divergence can be partially reduced by shifting the initial time point. Please check the descriptions in the **2th paragraph of the Data sources section of the Materials and methods part**. Because the dengue fever epidemic season in Guangdong province is mainly from August to October, and the epidemic peak is generally reached in October (please refer to: Li R, et.al. Climate-driven variation in mosquito density predicts the spatiotemporal dynamics of dengue. PNAS, 2019), the strategy of shifting the initial time point to the 19th week was also proposed in previous studies, which could partly avoid the filtering divergence caused by dealing with too many zero-inflated data in the earlier stages of data assimilation (please refer to: Chen Y, et.al. An ensemble forecast system for tracking dynamics of dengue outbreaks and its validation in China. PLoS Comput Biol. 2022). Based on your comment, we had revised the manuscript to make it more accurate. Please check them in **the Retrospective Forecasts section, the Materials and methods part**. Thank you for the valuable comments.

**2) Comment:** "The author use real-time forecasting might not be appropriate term because you deal with weekly data. You need to define the meaning of “real time” in your study. "

**Response:** Dear Professor, we feel sorry for our poor language expressions that made you misunderstood. We have revised the descriptions you mentioned to "retrospective forecasts", "weekly real-time ensemble predictions" or "reliable ensemble forecasts" in the revised manuscript. These similar expressions have also used in many previous studies (please refer to: Shaman, J.et al. Forecasting seasonal outbreaks of influenza. PNAS. 2012; Shaman, J.et al. Real-time influenza forecasts during the 2012–2013 season. Nat Commun. 2013). Thank you for the valuable comments.

**3) Comment:** "Since the model can give more accurate forecasting results about “peak time”, “peak intensity” and “total number” of cases. I was wondering to know whether the model can be beneficial to the dengue early warning system?"

**Response:** Dear Professor, thank you very much. We really thank you for giving us this opportunity to clarify the issue. In fact, our model is able to predict dengue epidemic in advance. Actually, we used the indicators including peak time, peak intensity and total number of cases to assess the performance of the model established in our study. These indicators were also proposed in previous studies (please refer to: Pei S et al. Forecasting the spatial transmission of influenza in the United States. PNAS. 2018; Shaman, J.et al. Real-time influenza forecasts during the 2012-2013 season. Nat Commun. 2013). These indicators allow us to assess the model's ability to forecast forward. According to the analysis results of the indicators, the dengue epidemic can be accurately predicted up to 10 weeks in advance (please see Fig 4B). Besides, at the time point of week 10, the model was used to generate predictions of the overall incidence of dengue fever in each epidemic season, as shown in Figs. S8 and S14. We performed 300 ensemble sub-models to achieve the forecast results. Corresponding 95% prediction intervals were also reported. Observations are generally contained within the prediction interval bands. Overall, we can see that the proposed model well predicts the dynamics of dengue epidemics in most cities. We feel sorry for our poor language expressions that made you misunderstood. We have revised some unclear expressions. Please check them in **the Abstract part, and the last paragraph of the Discussion part**. Thank you for the constructive comments.

**4) Comment:** "Regarding the 3rd limitation mentioned in the discussion, is it possible to develop sub-models to include either climate or mobility data only? If it doable, you can understand which parameters play more important role in your model."

**Response:** Dear Professor, the comments are insightful. We really thank you for giving us this opportunity to explain the issue here. The main purpose of this present study was to establish a forecast model with improved performance by accounting for the spatial co-variability of the transmission dynamics or levels of local susceptibility. In fact, we initially performed an empirical dynamics modeling framework to identify drivers for transmission dynamics of dengue fever. By adopting this approach, we aimed to determine which parameters play more important roles in the model. The method is proposed to identify the drivers of disease in previous studies (please refer to: Sugihara G et al. Detecting causality in complex ecosystems. Science. 2012; Nova N. et al. Susceptible host availability modulates climate effects on dengue dynamics. Ecol Lett. 2021). In our study, we found that the factors including mosquito oviposition index, ambient temperature, and population migration were important drivers for dengue dynamics. Please check relevant descriptions in **the 1st paragraph of the Result part as well as Fig. S6**. In addition, we would like to explain to you that the model constructed in this study belongs to the metapopulation network in essence, which is connected by the flow of migration among different spatial units. At present, there are some methodological issues for analyzing which factors play a more important role in the proposed metapopulation network. We have discussed the issue in **the 3rd paragraph of the Discussion part**. Please check it.

**5) Comment:** "No page and line number make it difficult to pinpoint questions."

**Response:** Dear Professor, thank you very much. We have added page and line numbers to make it easy to pinpoint questions.

**6) Comment:** "The vector’s name should write in this way: Aedes albopictus and Aedes aegypti".

**Response:** Dear Professor, we have revised the word errors according to your suggestion. Please check it in **the 1st paragraph of the Introduction part**.

**To Reviewer #2:**

Dear professor, we really thank you for your encouragement that "the authors present a novel method for more accurately tracking dengue virus disease dynamics by integrating a metapopulation network contact structure based on human mobility data inferred from cell phone use data, temperature, precipitation, and mosquito population dynamics. They utilize an ensemble-adjusted Kalman filter algorithm for data assimilation. Overall, the manuscript presents important findings on dengue analytic tools that will likely prove relevant in other geographic contexts and other disease contexts". Although you think our research has some important findings, you also pointed out that some issues on the objectives and contributions need to be clarified in order to make it clearer. We have carefully revised the manuscript according to your suggestions. We hope the revisions will make you feel appropriate and finally be approved by you.

**1) Comment:** "However, the objectives of the study and its novel contribution are not well described. More clarity on the relative contribution of this study and the implications for infectious disease dynamics modeling based on an understanding of the literature would strengthen this article's candidacy for publication. The first sentence of the discussion state's the main purpose of the research, but it is not clear how much of this purpose has been accomplished by previous studies, and this statement of purpose should come sooner in the manuscript. "

**Response:** Dear Professor, thank you very much for giving us the opportunity to elaborate on this issue. First, in our previous study, we proposed an SIR-EAKF model and confirmed that it could well capture the trend of dengue epidemic dynamics in population (please refer to Chen Y et al. An ensemble forecast system for tracking dynamics of dengue outbreaks and its validation in China. PLoS Comput Biol. 2022). However, there are still some issues that we haven't addressed in the previous work. For example, spatial diffusion is vital for understating the evolution of the complex, dynamical transmission of mosquito-borne diseases in the real world, and the spatial spread of infectious diseases is often caused by population movement (please refer to: Sanna et al. Spatial and Temporal Characteristics of 2014 Dengue Outbreak in Guangdong, China. Sci Rep. 2018; Rivera et al. Travel-Associated and Locally Acquired Dengue Cases-United States, 2010–2017. MMWR Morb Mortal Wkly Rep. 2020). Therefore, in this present study, we developed a forecast system incorporating population movement data into the proposed SIR-EAKF to more accurately predict the spatial spread of dengue virus. This present study represents the first attempt by establishing such a metapopulation network by connecting each city based on human mobility to track the spatial spread dynamics of dengue virus in the study area with high dengue prevalence. The metapopulation network provides reliable and accurate predictions that will help governments adopt more precise control strategies for dengue epidemics. We feel sorry that the objectives of the study and its novel contribution are not well described. Based on your comments, we have made the statement of purpose come sooner in the manuscript and added more descriptions in **the 2nd and 3rd paragraphs of the Introduction part and the 1st paragraph of the Discussion part.** Please check them. Thank you very much.

**2) Comment:** "It is also unclear if data and code will be made available, making the reproducibility of the study difficult."

**Response:** Dear Professor, thank you very much. The dengue surveillance data used in this study cannot be publicly available by uploading as supporting information due to the current confidentiality agreement on infectious disease surveillance data in China. In fact, the data are deposited in an appropriate repository. However, people can request access to the data from the National Population Health Science Data Sharing Platform. Therefore, for data availability, we have added the statement that “To protect the privacy of study participants, primary data are not publicly available. To access these data and/or to seek permission for its use, please contact the Data-center of China Public Health Science (https://www.phsciencedata.cn/Share/index.jsp) or email data@chinacdc.cn” in the manuscript submission system. Please check it. Thank you for your understanding.

**3) Comment:** "The first sentence says predicting the magnitude and timing of disease outbreaks is critical. But is that what this study really does? Does it predict where, when, and how bad novel outbreaks will be? Or does it rather improve accuracy of final epidemic size and when the epidemic will inflect, given outbreaks? Predicting the temporal peak of the epidemic is not the same as timing writ large."

**Response:** Dear Professor, thank you. We feel sorry about the inaccurate expression, and really agreed with your comment that "predicting the temporal peak of the epidemic is not the same as timing writ lage". It should be accurately expressed as "predicting the magnitude and the temporal peak of the epidemic of individual local outbreaks is critical for infectious disease control". In this study, the established spatial metapopulation model has the improved performance for predicting the epidemic size and temporal peak of dengue. Overall, we found that by assimilating retrospective surveillance data from multiple locations, our proposed method has enhanced prediction accuracy up to 100%, 75%, and 83.3% in terms of peak week, peak intensity and total incidence, respectively. According to your comment, we have revised corresponding descriptions in **the 1st sentence of the Abstract part, and the 1st sentence of the Discussion part**. Thank you very much.

**4) Comment:** "the authors conclude the abstract with asserting that the paper provides a foundation for forecasting mosquito-borne infectious diseases. Is this claim supported? Will this model work with malaria or other mosquito-borne diseases that utilize different mosquito vectors with different habits/preferences? Aedes mosquitoes are importantly different than Anopheles.

**Response:** Dear Professor, thank you very much. We really thank you for giving us this opportunity to clarify the issues. Previous studies have validated that the traditional SIR model coupled with the SIR-EAKF algorithm can be calibrated to the observed data stream and generates real-time predictions of infectious diseases, such as West Nile virus (WNV), Ebola, influenza, and other respiratory viruses (please refer to DeFelice NB et al. Ensemble forecast of human West Nile virus cases and mosquito infection rates. Nat Commun. 2017; Shaman J et al. Inference and forecast of the current west African Ebola outbreak in Guinea, Sierra Leone and Liberia. PLoS Curr. 2014; Pei S et al. Optimizing respiratory virus surveillance networks using uncertainty propagation. Nat Commun. 2021; Anderson JL. An Ensemble Adjustment Kalman Filter for Data Assimilation. Mon Wea Rev. 2001). The present study extends the traditional SIR-EAKF model to the spatial scale, enabling it to analyze the spatial spread of dengue virus. Therefore, this model works with other mosquito-borne diseases, although the diseases utilize different mosquito vectors with different habits. Actually, the model is a universal framework, as long as the parameters of mosquito vectors are adjusted accordingly. We really agree with your comment that “Aedes mosquitoes are importantly different than Anopheles”. However, the metapopulation network proposed in this study has not yet been applied to predicting the dynamics of other mosquito-borne infectious diseases. Our study represents the first attempt to establish such a framework for the prediction of dengue fever. Considering your comments and the major contribution of our study, we think that it should be expressed more accurately. Therefore, we have stated it as ‘dengue fever’ rather than ‘the mosquito-borne infectious diseases’ in the revised manuscript according to your suggestion. Please check it **in the Abstract part**. Thank you.

**5) Comment:** "The statement that human mobility increases the rapidity of geographic spread of dengue does not logically lead to the following statement that 'therefore, the establishment of models... is necessary for epidemic preparedness.' That would depend on how such models are used."

**Response:** Dear Professor, thank you for this precise comment. This original sentence is illogically expressed and is prone to misunderstanding. We have revised the sentence as "the increased possibility of individual exposure to disease vectors due to human travel allowed for the rapid spread of dengue virus to new susceptible populations, ultimately resulting in severe febrile illness and substantial disease burden. Therefore, the integration of information on human movement into infectious disease prediction model will contribute to the prevention and control of infectious diseases". We have revised the statement based on your comment in **the Author summary part**. Please check it.

**6) Comment:** "The authors state that 'this study developed and validated a forecasting system for predicting the spatial spread of dengue.' However, the model has not been used in predicting and forecasting, except retrospectively. This statement should include the timeframe of the data utilized."

**Response:** Dear Professor, thank you very much for giving us the opportunity to correct this mistake. The statement should be accurately expressed as “retrospectively predict”. We have carefully added the timeframe of the data utilized in the revised manuscript according to your suggestion. Please check them in the revised manuscript. We really thank you for the accurate comments.

**7) Comment:** "Please state the estimate of the economic loss, rather than use the subjective term 'enormous."

**Response:** Dear Professor, thank you for your careful. According to your suggestion, we have added the estimate of the global economic loss and relevant reference in **line 121 of the Introduction part**. Please check it. Thank you.

**8) Comment:** "'Previous studies have proposed...'. Only one study is cited here. "

**Response:** Dear Professor, thank you for this insightful comment. We have added the references in **the 2nd sentence of the Introduction part** according to your suggestion. Please check it.

**9) Comment:** "It is not clear what, precisely, has been already done in the literature. Can you state more precisely what the literature has studied so far and what specifically is novel about this approach? It seems that previous studies have looked at time series data, but others have also used human mobility. And cell phone use is likewise not novel. Is the novelty in this study the use of the Kalman filter? Or are the metapopulation structures also novel? If, for example, the sole novelty of the study is the inclusion of the Kalman filter, then when testing its performance compared to previous methods, it would be interesting to see the same model structure without the use of the filter algorithm. How do the constituent parts of the model explain or influence disease dynamics and can we rank-order their influence? "

**Response:** Dear Professor, we really appreciate your constructive comments and would like to explain the issue to you clearly. The responses are divided into the following points:

First, we should acknowledge that your comment is very insightful. In fact, our previous study has compared and assessed the performance of model structure with the use of the Kalman filter and that without the inclusion of the filter algorithm (please refer to: Chen Y et al. An ensemble forecast system for tracking dynamics of dengue outbreaks and its validation in China. PLoS Comput Biol. 2022). Our previous study showed that the SIR-EAKF model could well capture the trend of the dengue epidemic trajectory. However, this model has not been able to predict spatial epidemic dynamics of dengue. The model only considers the time trend of dengue in a single geographic location. As previous research has pointed out, spatial diffusion is vital for understating the evolution of real-world complex dynamical transmission of mosquito-borne diseases, and human mobility plays an important role in the spatial spread of diseases (please refer to: [1] Sanna M et al. Spatial and Temporal Characteristics of 2014 Dengue Outbreak in Guangdong, China. Sci Rep. 2018; [2] Rivera A et al. Travel-Associated and Locally Acquired Dengue Cases—United States, 2010–2017. MMWR Morb Mortal Wkly Rep. 2020). Therefore, in order to more accurately predict the spatial spreads of dengue, we constructed such a novel metapopulation network based on the SIR-EAKF, which can only predict dengue from a time series perspective. Overall, one particular contribution of this present study is that we introduced a spatial metapopulation model by connecting each city based on human mobility, and made predictions of dengue spatial transmission. The advent of the metapopulation networks improves performance by accounting for the spatial co-variability of the transmission dynamics or levels of local susceptibility. The model provides a way to facilitate the transition of dengue forecast from point to space and to incorporate it into decision-making. According to your suggestion, we have revised the manuscript to highlight the novelty and the contributions. Please check them in **the 2nd and 3rd paragraphs of the Introduction part and** **the 2nd paragraph of the Discussion part**.

Second, prior to establishing this model, we have also performed the analysis of which parameters play more important role in the model. We used the empirical dynamic modeling (EDM) method (please refer to: Sugihara G et al. Detecting causality in complex ecosystems. Science. 2012; Nova N. et al. Susceptible host availability modulates climate effects on dengue dynamics. Ecol Lett. 2021) to identify the drivers for the dynamics of dengue epidemics. We found that the parameters including mosquito oviposition index, ambient temperature, and population migration are important drivers for dengue dynamics. Please check **Fig. S6 and the 1st paragraph of the Result part**. The estimation and identification of the importance of parameters in the metapopulation network in the strict mathematical sense is one of the urgent methodological problems, which needs to be further studied in the future. We have discussed the issue you mentioned above in **the 3rd paragraph of the Discussion part**. Thank you very much.

**10) Comment:** "Why were small outbreaks excluded? Can the model not predict small outbreaks too? Including small outbreaks seems important if the ultimate goal is accurate forecasting for epidemic preparedness."

**Response:** Dear Professor, we feel sorry about the poor language in the previous version of manuscript that makes you confused. The weekly dengue fever cases in each city of Guangdong province in 2018-2020 were shown in Figs. S1 and S2. As shown, the annual number of cases in these excluded cities (such as only 1 case in 2019 in Shanwei city and 6 cases in 2019 in Shaoguan city) is very small. According to the time series characteristics of infectious diseases, they are sporadic cases, but not “small outbreak”. From the perspective of time series analysis, these sporadic cases can basically be treated as random numbers (such as only 1 case in 2019 in Shanwei city, etc.). The number of cases is very sparse and it is difficult to model the disease transmission using existing methods. However, we feel that your comment is quit important, and we have tried to elaborate this issue. Please check it in **the 2nd paragraph of the Materials and methods part**.

**11) Comment:** "Is 'eggs / effective eggs' a list or an equation?"

**Response:** Dear Professor, thank you for this comment. We originally wanted to use this notation (/) to express the meaning of ‘or’. We would like to show the calculation of the mosquito oviposition index (MOI), established by the literature (Rozilawati H et al. Seasonal abundance of Aedes albopictus in selected urban and suburban areas in Penang, Malaysia. Trop Biomed. 2007). Based on your comment, we have revised the corresponding statements. Please check it in **the 3rd paragraph of the Materials and methods part**. Thank you for the valuable comment.

**12) Comment:** "Multiple formula are mentioned but not delineated. Can you delineate the formula used for MOI and dengue virus reproduction associated with temp? We need to have the math in the paper in order to understand how the model is constructed."

**Response:** Dear Professor, we really thank you for the constructive comment. We are very sorry for our earlier oversight in the manuscript submitted in the first round. The relationship between ambient temperature and population transmission rate was represented by a piecewise linear function based on Liu et al. (please refer to Liu Z et al. Temperature Increase Enhances Aedes albopictus Competence to Transmit Dengue Virus. Front Microbiol. 2017). The function is listed as follows:

$$f(T)=\left\{\begin{matrix} 0 & \text{T}\leq18 \\ (T-18)\times8/3 & 18<\text{T}\leq23 \\ 40/3+(T-23)\times16/3 & 23<\text{T}\leq28 \\ 40+(T-28)\times20/3 & 28<\text{T}\leq32 \\ 200/3 & \text{T>32} \end{matrix}\right.$$

where $T$ is the daily ambient temperature, and $f(T)$ is the population transmission rate. According to your suggestion, we have added corresponding descriptions about the formula. Please check them in **the 4th paragraph in the Data sources section,** **the Materials and methods part**. Thank you very much.

**13) Comment:** "If China Unicom data is used, and that represents only 19% of market share, how is mobility writ large scaled up? Do you assume all cell phone carriers have the same mobility? What about people without cell phones?"

**Response:** Dear Professor, thank you very much for this valuable comment. We really appreciate this opportunity to explain the issue to you. First, we would like to explain to you that the high-resolution data of population movement used in this study is the best available at present in China, mainly for better security of mobile phone user data in the country. Second, in order to reduce the bias caused by insufficient data of population movement, an approach by incorporating an adjustable parameter *θ* (random movement ratio) has been performed to simulate real human mobility compensating in our model. This method has also been proposed by Pei and his colleagues (please refer to: Pei S et al. Forecasting the spatial transmission of influenza in the United States. PNAS. 2018). In particular, we simulate irregular movement besides population mobile data. These individuals follow the Markov process and circulate among subpopulations, resulting in population exchange among all subpopulations (please refer to: Rivera A et al. Travel-Associated and Locally Acquired Dengue Cases-United States, 2010-2017. MMWR Morb Mortal Wkly Rep. 2020). Although these measures have been taken to control bias, we agree that the inevitable limitations about the data of population movement should be noted. We have discussed the limitation in **the 4th paragraph of the Discussion part**. Please check it. Thank you very much.

**14) Comment:** "How have mobility restrictions due to COVID-19 affected mobility and migration in the 2020 data and modeling?"

**Response:** Dear Professor, thank you for this comment. We would like to explain it to you here. The retrospective prediction of the season included in this study ended in February 2020. At that time, the outbreak of COVID-19 has only occurred in Wuhan city of Hubei province, and the outbreak has not yet spread to Guangdong (the study area of our study). Please check the retrospective forecast seasons marked by grey areas in Fig. S4. In fact, the results of this study are less likely to be influenced by COVID-19. We really appreciate your insightful comment.

**15) Comment:** "The specifics of the metapopulation are not given. Consider a map or figure to illustrate how the metapopulation is structured and how it interacts between constituent nodes. I would also like to see a theoretical figure showing the inclusion of different modules (e.g., the metapopulation, mosquito population, temp, etc.) to better understand visually how the model works."

**Response:** Dear Professor, thank you very much. According to your suggestion, we have added **Figure 2** in the revised manuscript. In addition, we have added necessary descriptions in **the 1st paragraph of the Methods part.** Please check them.

**Figure 2.** **Model structure.** Blue and black rectangles represent mosquito and human subpopulations, respectively. The yellow compartments represent the proliferation of dengue virus in mosquitoes at the suitable temperature. The right chart shows the human movements structure of the crowd among the various subpopulations.

**16) Comment:** "What are the assumptions of the EAKF? And are these assumptions met in this case? Is there an assumption of independence, for example, and are these variables independent?"

**Response:** Dear Professor, thank you for this insightful comment. In fact, the Kalman filters assume that both the prior distribution and likelihood are Gaussian, thus the distributions can be fully parameterized by the first two moments (mean and covariance) (please refer to: Pei S et al. Counteracting structural errors in ensemble forecast of influenza outbreaks. Nature communications. 2017). In addition, our previous study has shown that the assumptions of EAKF are met, and the data assimilation method has been proven to deal with dengue cases (please refer to: Chen Y, et.al. An ensemble forecast system for tracking dynamics of dengue outbreaks and its validation in China. PLoS Comput Biol. 2022). According to your suggestion, we have added necessary descriptions of the model assumptions. Please check it in **the last paragraph of the Method part of the “Supporting Information” file**.

**17) Comment:** "Consider utilizing different labeling, so we can see how the graphics are paired (e.g. A1, A2, B1, B2...). Further, given the similarities (theta) of all graphs on the left v. right, consider a table of graphics."

**Response:** Dear Professor, thank you for this insightful comment. According to your suggestion, we have revised the figure in order to better show the results. Please check it. Thank you.

**18) Comment:** "Any mentions of significance should cite the statistical method used to determine."

**Response:** Dear Professor, thank you very much. We have added the description of the statistical method. Please check it in **the 4th paragraph of the Results part.**

**19) Comment:** "Rather than report 'better' or 'worse,' consider reporting the magnitude of the differentials and analyzing for significance."

**Response:** Dear Professor, your comment is reasonable and understandable. Based on your suggestions, we have added the results of statistical significance for prediction performance. Please check it in **Table 1**.

**20) Comment:** "More can be done in the discussion to explore the study's contribution to the literature."

**Response:** Dear Professor, thank you very much. We have discussed the contribution of our study according to your suggestion. Please check them in **the 2nd paragraph of the Discussion part**.
