# Supplementary material for: Dengue transmission dynamics prediction by combining metapopulation networks and Kalman filter algorithm
Source: PLoS Negl Trop Dis. 2023 Jun 7;17(6):e0011418. doi: 10.1371/journal.pntd.0011418 (PMC10281582; doi:10.1371/journal.pntd.0011418)
Supplement: Attachment — Submitted filename: Response to Reviewers.doc [file pntd.0011418.s019.doc]

**Manuscript Number:** PNTD-D-22-00643

**Title:** Dengue transmission dynamics prediction by combining metapopulation networks and Kalman filter algorithm

**Journal:** PLOS Neglected Tropical Diseases

Dear Professor Eric HY Lau and Robert Reiner,

Thank you very much for giving us the opportunity to revise our manuscript. We strongly endorse the three reviewers' suggestions, which have greatly improved our manuscript. Every change has been outlined and the responses to the comments have been made point by point. We have also expressed our sincere appreciation of your help in the Acknowledgment section of the manuscript. Our responses to the comments have been given in the next page.

In this round of revision, we have submitted the files as follows:

1. A cover letter (uploaded as a **"Cover Letter" file**) was uploaded.

2. A letter (uploaded as a **"Response to Reviewers" file**) containing a detailed list of our responses to the review comments and a description of the changes we have made was uploaded.

3. A marked-up copy with tracked changes denoting where the text has been changed (uploaded as a **"Revised Manuscript with Changes Marked" file**) was uploaded.

4. A clean revised manuscript (uploaded as a **"Manuscript" file**) was submitted.

5. All the figures were uploaded separately, and a supplementary material file (uploaded as a **"Supporting Information" file**) was also uploaded.

Yours sincerely,

Pi Guo

Department of Preventive Medicine, Shantou University Medical College

Email: pguo@stu.edu.cn

**To Editor:**

**1) Comment:** “The Authors are expected to address all the criticisms by all Reviewers. In particular, please address all comments from Reviewer #3 from the previous round, consider the impact of significant mobility change in early 2020 and clarify the metapopulation model (Reviewer #3). In additional to these comments, please address: EAKF may handle zero-inflated data, but the significant change in human mobility after the lockdown in late January 2020 was not captured in the model (e.g. suspension of transport across or within county/provinces, cancellation of cultural/sport events). This could be a study limitation. However, this period is expected to have limited dengue with or without COVID and should have a limited impact on the overall results.”

**Response:** Dear editor, thank you very much for giving us this opportunity to revise the manuscript. We really agree that the lockdown following the COVID-19 pandemic in 2020 resulted in a significant reduction in population movements. However, during the period from January to May 2020, the dengue fever season in Guangdong province had essentially ended and the next dengue outbreak had not yet occurred. We really agree with you that “this period is expected to have limited dengue with or without COVID and should have a limited impact on the overall results”. We strongly agree with you, and in order to present our results more rigorously, we have discussed the issue in the Discussion part of this revised manuscript as a limitation. Please check it in **the 4th paragraph of the Discussion part**. Thank you again.

**To Reviewer #3:**

Dear professor, we really thank you for your encouragement that "the authors have made major modifications in the revised manuscript". We really appreciate your insightful comments that "there are one problem that the author have explained irrationally, and there is some problems that the authors have not revised". We really cherish this opportunity and have carefully revised the manuscript according to your suggestions. We hope our revisions have made it more understandable for potential readers. Our responses to the comments have been arranged one by one in the following section.

**1) Comment:** " Both reviewers #2 and #3 comment how travel restrictions due to COVID-19 have affected the pattern of population movement in the modeling. The response of authors is that “the retrospective prediction of the season included in this study ended in February 2020. At that time, the outbreak of COVID-19 has only occurred in Wuhan city of Hubei province, and the outbreak has not yet spread to Guangdong (the study area of our study).” This response is unreasonable. Firstly, in lines 195 and 196, the dengue season is defined as the 19th week of the former year to the 20th week of the succeeding year. So the season included in this study should ended in May 2020 rather than February 2020. Secondly, Guangdong reported its first confirmed case of COVID-19 on January 20, and by February 29, the total number of confirmed cases had reached 1,349. So the opinion that “the outbreak has not yet spread to Guangdong” is wrong. Thirdly, due to the COVID-19 outbreak in China in 2020, all Chinese provinces issued travel restrictions in late January, including Guangdong Province. The authors are advised to reconsider this comment."

**Response:** Dear Professor, we really appreciate your rigorous comment. As you commented above, in our study the dengue season is defined as the 19th week of the former year to the 20th week of the succeeding year. Thus, the season included in this study should have ended in May 2020. It is true that the COVID-19 pandemic occurred during this study period and that population movement changed significantly due to lockdown measures. However, the Figure e1 below shows that there are very few dengue cases during the period from January to May 2020 in Guangdong province. Therefore, the dengue season defined in this study had essentially ended in May 2020. This approach of defining disease epidemic seasons according to epidemic intensity is mainly to better verify the predictive accuracy of the model. The approach is also widely proposed in the previous studies of influenza models (please refer to : Shaman J, et al. Real-time influenza forecasts during the 2012-2013 season. *Nature communications*. 2013). We can observe that during the periods outside the dengue season there were very few dengue cases. For example, in some weeks outside the dengue season there were only 7 or 8 cases. Therefore, it is a big challenge to accurately predict such a time series of almost random fluctuations. We are sorry for our careless response before. The opinion that “the COVID-19 outbreak has not yet spread to Guangdong” during the period from January to May of 2020 is wrong. Although China restricted travel in early 2020 in response to the outbreak of COVID-19, this period is expected to have limited dengue cases with or without COVID and should have a limited impact on the overall results. This viewpoint is also echoed by the editors. Please check the comments above in response to the editors. We strongly agree with you, and in order to present our results more rigorously, we have discussed the issue in the Discussion part of this revised manuscript as a limitation. Please check it in **the 4th paragraph of the Discussion part**.

**Figure e1. Weekly dengue fever cases (cross-sign) in each city of Guangdong Province in 2019-2020 season.**

**2) Comment:** "In subsection Metapopulation network, the metapopulation network model is problematic. In Equation (2), the authors ignored the infection caused by mosquitoes biting travelers from other subpopulations I_n^k."

**Response:** Dear Professor, thank you very much. We would like to explain the issue to you. Equation (1) and Equation (2) model the dynamics of dengue transmission in human and mosquito populations, respectively. Equation (1) have accounted for human infection caused by mosquitoes biting travelers from other subpopulations $I_{nH}^{k}$. Therefore, the infections $I_{nH}$ has included infected persons who arrived from other subpopulations at time t-1. When calculating the number of mosquitos in each compartment of equation 2, we only need to consider the local human infections at time t. These infections already incorporate those from other subpopulations at time t-1. The original notation ($I_{nH}^{n}$) might not be very clear, so we have revised it as $I_{nH}$.

Please check it in **the 3rd paragraph of the Metapopulation network section of the Materials and methods part.**

**3) Comment:** "In lines 302 and 303, “U was the dissemination rate constant over an outbreak”. This definition is a little confusing. Maybe it is more reasonable to define U as vertical infection rate."

**Response:** Dear Professor, thank you very much. We have revised the term according to your suggestion. Please check it in **the 3rd paragraph of the Metapopulation network section, the Materials and methods part.**

**4) Comment:** "In Equation (2), what are the values of \mu_b(t) and \tau(t)? Please explain them in detail."

**Response:** Dear Professor, thank you very much. We apologize for the unclear expression of the formula that caused confusion. The symbol $\mu_{b}(t)$ denotes the mosquito birth rate at time *t*, the symbol $\mu_{d}$ represents the constant mosquito death rate over an outbreak, the $\tau_{n}\left(t\right)$ is the population transmission rate at time t, the β0 is the basic contact rate between humans and mosquitoes, and $\tau_{n}\left(t\right)\beta_{0}$ is the contact rate between humans and mosquitoes at time *t*. The annual average time series of mosquito birth rate ($\mu_{b}(t)$) and population transmission rate ($\tau_{n}\left(t\right)$) were calculated, and incorporated in the compartmental model describing the dynamics of disease transmission. A time-varying $\mu_{b}(t)$ and a fixed death rate ($\mu_{d}$) can simulate the natural growth of mosquito population. The parameter $\tau_{n}\left(t\right)$ estimated by temperature in the model can explain the probability of successful transmission of dengue virus after a mosquito bite. In our previous study, we also set up similar parameters and verified their validity (please refer to: Chen Y, et.al. An ensemble forecast system for tracking dynamics of dengue outbreaks and its validation in China. *PLoS Computational Biology*. 2022). According to your comments, we have further made some revisions to make the statement more accurate. Please check it in **the 2nd and 3rd paragraphs** of **the Metapopulation network section, the Materials and methods part.** We hope the above explanation will shed some light on this issue.

**5) Comment:** "In lines 328 and 329, what do S_{nM}^k and I_{nM}^k mean? In line 365, what does N_{nM} mean? Do they mean that mosquito could move between cities? If so, it contradicts the assumption on line 296. Please explain them in detail."

**Response:** Dear Professor, thank you very much for the valuable suggestions. The numbers of susceptible mosquitoes ($S_{nM}^{k}$) and infected mosquitoes ($I_{nM}^{k}$) should be correctly rewritten as $S_{nM}$ and $I_{nM}$, representing the local populations of susceptible and infected mosquitoes, respectively. Please check them in **the 2nd paragraph of the Parameter Inference section of the Materials and methods part, and the 1st paragraph of the Retrospective Forecasts section of the Materials and methods part.** Thank you again for your important comments.
